# Supplementary material for: CNVpytor: a tool for copy number variation detection and analysis from read depth and allele imbalance in whole-genome sequencing
Source: Gigascience. 2021 Nov 18;10(11):giab074. doi: 10.1093/gigascience/giab074 (PMC8612020; doi:10.1093/gigascience/giab074)
Supplement: giab074_GIGA-D-21-00151_Revision_3 [file giab074_giga-d-21-00151_revision_3.pdf]

GigaScience

CNVpytor: a tool for CNV detection and analysis from read depth and allele imbalance in whole genome sequencing

--Manuscript Draft--

**Manuscript Number:**	GIGA-D-21-00151R3
**Full Title:**	CNVpytor: a tool for CNV detection and analysis from read depth and allele imbalance in whole genome sequencing
**Article Type:**	Technical Note
**Funding Information:**	National Cancer Institute (U24CA220242) — Dr. Alexej Abyzov
**Abstract:**	Background: Detecting copy number variations (CNVs) and copy number alterations (CNAs) based on whole genome sequencing data is important for personalized genomics and treatment. CNVnator is one of the most popular tools for CNV/CNA discovery and analysis based on read depth (RD).<br>Findings: Herein, we present an extension of CNVnator developed in Python -- CNVpytor. CNVpytor inherits the reimplemented core engine of its predecessor and extends visualization, modularization, performance, and functionality. Additionally, CNVpytor uses B-allele frequency (BAF) likelihood information from single nucleotide polymorphism and small indels data as additional evidence for CNVs/CNAs and as primary information for copy number neutral losses of heterozygosity.<br>Conclusions: CNVpytor is significantly faster than CNVnator—particularly for parsing alignment files (2 to 20 times faster)—and has (20-50 times) smaller intermediate files. CNV calls can be filtered using several criteria, annotated, and merged over multiple samples. Modular architecture allows it to be used in shared and cloud environments such as Google Colab and Jupyter notebook. Data can be exported into JBrowse, while a lightweight plugin version of CNVpytor for JBrowse enables nearly instant and GUI-assisted analysis of CNVs by any user. CNVpytor release and the source code are available on GitHub at https://github.com/abyzovlab/CNVpytor under the MIT license.
**Corresponding Author:**	Alexej Abyzov, Ph.D.<br>Mayo Clinic: Mayo Clinic Minnesota<br>Rochester, Minnesota UNITED STATES
**Corresponding Author Secondary Information:**	
**Corresponding Author's Institution:**	Mayo Clinic: Mayo Clinic Minnesota
**Corresponding Author's Secondary Institution:**	
**First Author:**	Milovan Šuvakov
**First Author Secondary Information:**	
**Order of Authors:**	Milovan Šuvakov
	Arijit Panda
	Colin Diesh
	Ian Holmes
	Alexej Abyzov
**Order of Authors Secondary Information:**	
**Response to Reviewers:**	We have addressed suggested editorial points:<br>- Supplemental material is updated.<br>- biotools ID is updated.<br>- GigaDB dataset is added in references and cited from "data availability" paragraph.
**Additional Information:**	

Powered by Editorial Manager® and ProduXion Manager® from Aries Systems Corporation

Question	Response
Are you submitting this manuscript to a special series or article collection?	No
**Experimental design and statistics**<br><br>Full details of the experimental design and statistical methods used should be given in the Methods section, as detailed in our Minimum Standards Reporting Checklist. Information essential to interpreting the data presented should be made available in the figure legends.<br><br>Have you included all the information requested in your manuscript?	Yes
**Resources**<br><br>A description of all resources used, including antibodies, cell lines, animals and software tools, with enough information to allow them to be uniquely identified, should be included in the Methods section. Authors are strongly encouraged to cite Research Resource Identifiers (RRIDs) for antibodies, model organisms and tools, where possible.<br><br>Have you included the information requested as detailed in our Minimum Standards Reporting Checklist?	Yes
**Availability of data and materials**<br><br>All datasets and code on which the conclusions of the paper rely must be either included in your submission or deposited in publicly available repositories (where available and ethically appropriate), referencing such data using a unique identifier in the references and in the “Availability of Data and Materials” section of your manuscript.	Yes

Have you have met the above requirement as detailed in our Minimum Standards Reporting Checklist?	

Powered by Editorial Manager® and ProduXion Manager® from Aries Systems Corporation

Manuscript

Click here to
access/download;Manuscript;CNVpytor_manuscript.docx

CNVpytor: a tool for CNV detection and analysis from read depth and allele imbalance in whole genome sequencing

Milovan Suvakov[1,2], Arijit Panda[1,2], Colin Diesh[3], Ian Holmes[3], Alexej Abyzov[1,2,*]

[1]Department of Quantitative Health Sciences, Mayo Clinic, Rochester, MN

[2]Center for Individualized Medicine, Mayo Clinic, Rochester, MN

[3]Department of Bioengineering, University of California, Berkeley, USA

*Correspond author. Email: abyzov.alexej@mayo.edu

Keywords: copy number variations, copy number alternations, whole genome sequencing, Python

Abstract

Background: Detecting copy number variations (CNVs) and copy number alterations (CNAs) based on whole genome sequencing data is important for personalized genomics and treatment. CNVnator is one of the most popular tools for CNV/CNA discovery and analysis based on read depth (RD).

Findings: Herein, we present an extension of CNVnator developed in Python -- CNVpytor. CNVpytor inherits the reimplemented core engine of its predecessor and extends visualization, modularization, performance, and functionality. Additionally, CNVpytor uses B-allele frequency (BAF) likelihood information from single

nucleotide polymorphism and small indels data as additional evidence for CNVs/CNAs and as primary information for copy number neutral losses of heterozygosity.

Conclusions: CNVpytor is significantly faster than CNVnator—particularly for parsing alignment files (2 to 20 times faster)—and has (20-50 times) smaller intermediate files. CNV calls can be filtered using several criteria, annotated, and merged over multiple samples. Modular architecture allows it to be used in shared and cloud environments such as Google Colab and Jupyter notebook. Data can be exported into JBrowse, while a lightweight plugin version of CNVpytor for JBrowse enables nearly instant and GUI-assisted analysis of CNVs by any user. CNVpytor release and the source code are available on GitHub at https://github.com/abyzovlab/CNVpytor under the MIT license.

Introduction

The continuous reduction of cost made the whole genome sequencing (WGS) to be widely used in different research projects and clinical applications. Consequently, many approaches for processing, analyzing, and visualizing WGS data have been developed and are being improved. Detection and analysis of copy number variations (CNVs) based on WGS data is one of them. Research directions related to the cancer genomics, single-cell sequencing, and somatic mosaicism create huge amounts of data and demands for processing on the cloud that require further improvements of CNV callers, moving to parallel processing, better compression, modular architecture, and new statistical methods.

CNVnator is a method for CNV analysis based on read depth (RD) of aligned reads. It was determined to have high sensitivity (86%-96%), low false-discovery rate (3%-20%), and high genotyping accuracy (93%-95%) for germline CNVs in a wide range of sizes from a few hundred base pairs to chromosome size events [1-5]. Since its development a decade ago, the tool has been widely used in different scientific areas by researchers around the world for detection of CNVs in a variety of species with different genome sizes: bacteria [6], fungi [7], plants [8-10], insects [11], fish [12], birds [13], mammals [14-17] and humans [2, 18-20]. It has been used to discover somatic variations in cancer and disease studies [21] and to find mosaic variants in human cells [22]. Although CNVnator was developed to detect germline CNVs, it is well-suited to discover copy number alteration (CNAs) present in a relatively high (>50%) fraction of cells, such as somatic alteration found in cancers. It was not, however, designed for nor capable of aiding analysis of copy number neutral changes.

Here we describe CNVpytor, a Python extension of CNVnator. CNVpytor inherits the reimplemented core engine of CNVnator and extends visualization, modularization, performance, and functionality. Along with RD data, it enables consideration of allele frequency of single nucleotide polymorphism (SNP) and small indels as an additional source of information for the analysis of CNV/CNA and copy number neutral variations. Along with RD data, this information can be used for genotyping genomic regions and visualization.

Results

Analysis of RD signal

CNVpytor inherits the RD analysis approach developed in CNVnator [1]. Briefly, it consists of the following steps: reading alignment file and extracting RD signal, binning RD signal, correcting the signal for GC bias, segmenting the signal using the mean-shift technique, and calling CNVs (**Fig. 1**). RD signal can be parsed from BAM, SAM, or CRAM alignment files and is counted in 100 bp intervals, resulting in a small footprint of intermediate .pytor files in HDF5 format (**Table 1**). Because of using the pysam [23] library for parsing, this step (the most time-consuming one) is parallelized and can be conducted very efficiently, particularly in comparison with the older tool (**Table 1**). The binning step integrates RD over larger bins that are limited to multiples of initially stored 100 bp bins. Bin size can be adjusted by user depending on application. Depending on sequence coverage recommended minimal bin size is provided in Supplement (**Table S1**). Next, technical biases in the read depth signal that are correlated with GC content (so-called GC biases) are removed using GC correction procedure. For the human reference genomes GRCh37 and GRCh38 per bin, GC content is pre-calculated and supplied as a resource with the CNVpytor package. For other genomes, GC content can be calculated during runtime from a provided FASTA file or precalculated and added to the CNVpytor resource for future use. Once information about read coverage (and variants, see below) is extracted from an alignment file, the following analysis steps take place (i.e., read input and write output) with the same file. As a result, histograms for each processed bin size and information about CNV calls, including coordinates, different statistics, and p-values are all stored in one .pytor file and can be extracted into Excel (TSV file) or a VCF file.

Figure 1. Schematics of core algorithm and data processing steps. (A) Read depth analysis steps include parsing alignment file, calculating and storing read depths in 100 bp intervals, binning using user-specified bin size, correcting RD for GC bias, segmenting by mean-shift, and calling CNVs. (B) B-allele frequency analysis steps include reading variant file storing the data about SNPs and small indels, filtering variants using strict mask, calculating BAF for heterozygous variants (HETs), and calculating likelihood function for bins. For CNVs, BAF signal splits away from value 0.5 expected for HETs. (C) Distribution of the variant allele frequency for all variants and variants within strict mask as defined by the 1000 Genomes Project. Black line shows fit by gaussian distribution. (D) An example of RD depending on GC within bin. Statistics of RD signal within bins of the same percentage of GC content is used to correct for GC bias in the signal. White line represents average RD level for bins with given GC content. (E) An example of RD and BAF signals for a germline duplication in NA12878 sample (raw RD signal is in grey, GC-corrected RD signal is in black, brighter color of BAF likelihood corresponds to higher values of the likelihood).

Table 1. Efficiency of parsing alignment file on modern computers in relation to sequencing coverage and engaged number of CPU cores.

Sequencing coverage of the human genome	Parsing time				File size			
	CNVnator	CNVpytor			CNVnator	CNVpytor		
		4 cores	8 cores	23 cores		RD parsing	BAF parsing	With 1 & 10 kbp bins
5X	10 min	5 min	3 min	<2 min	1 Gb	18 Mb	20 Mb	250 Mb
30X	1 h	28 min	18 min	10 min	1.5 Gb	19 Mb	20 Mb	250 Mb
100X	3.3 h	1.5 h	1 h	33 min	2 Gb	20 Mb	20 Mb	250 Mb

Analysis of variant data

A novel feature of CNVpytor is the analysis of information from SNPs and small indels imported from a VCF file. An imbalance in the number of haplotypes can be measured using allele frequencies traditionally referred to as B-allele frequency (BAF) [24-27]. The main advantage of using BAF compared to RD is that BAF values do not require normalization and are distributed around 0.5 by binomial distribution for heterozygous variants (HETs). Additionally, BAF is complementary to RD signal, as it changes for copy number neutral events such as loss of heterozygosity. However, BAF dispersion can be measured incorrectly due to systematic misalignment particularly in repeat regions, incomplete reference genome, or site-specific noise in sequencing data. To mitigate this issue, we filtered out HETs in the fraction of genome that is inaccessible to short read technologies, as defined by the strict mask from the 1000 Genomes Project [28]. Such filtering removes almost all HETs with outlier values of BAF, while values for the retained variants closely follow binomial distribution (**Fig. 1C**). To integrate BAF information within bins, CNVpytor calculates the likelihood function that describes an imbalance between haplotypes (see **Methods**). Currently, BAF information is used when genotyping a specific genomic region where, along with estimated copy number, the output contains the average BAF level and two independent p-values calculated from RD and BAF signal. Variant data can also be plotted in parallel with RD signal (**Fig. 2**). Same as for RD signal, binned information calculated from variants is stored in and can be extracted from .pytor file.

Figure 2. BAF signal corroborates and complements RD signal. Example of CNVpytor *region* plots produced for deletion (left), duplication (middle), and CNN-LOH (right) for NA12878 sample. Within the coordinates of heterozygous deletion, there is a 50% drop in RD signal and a loss in heterozygosity in BAF signal (i.e., no heterozygous SNPs in the region). Duplication of one haplotype results in the increase of RD signal by 50% and in a split in VAF distribution of SNPs and a split in BAF likelihood function. In the CNN-LOH region, few reliable heterozygous SNPs are detected while RD signal does not change. Likelihood function values are normalized to maximal across the range.

Figure 3. CNVpytor workflow. Steps used in data processing. On the left: reading RD data from alignment file, creating histograms, segmentation, calling CNVs. On the right: reading SNP and indel data from VCF file, filtering variants using strict mask, calculating histograms and likelihood function. In the middle: alternatively, if alignment file is not available RD signals can be calculated from variant data (green arrows). Visualization using both RD and/or BAF data can be done from an interactive command line interface or automatically by running script file.

Running CNVpytor

CNVpytor is to be run in a series of steps (**Fig. 3)**. For enhanced flexibility, RD and BAF processing workflows proceed in parallel. In this way each workflow can be run at different times or even on different computers. For example, data parsing steps can be run on a cloud where data (i.e., alignment files) is accessible, resulting in less than 25 Mb .pytor files that then can be copied to local computer/cluster where the remaining calculation steps will be performed. If necessary, a user can run additional calculations (e.g., conduct processing with different bin size) using the same .pytor file in the future, allowing for further flexibility in data analysis.

Routine processing steps can be followed by CNV visualization and analysis in the Viewer session, which can be interactive or hands-off. Implementation of interactive mode is inspired by a Linux shell with tab

completion and with a help page similar to the man pages. In this mode a user can instantly make various visualizations, preview and filter CNV calls, annotate calls, create joint calls across multiple samples, and genotype specified regions. The viewer does not save results into the .pytor file, and outputs are printed and plotted on the screen or exported to an output file(s). Hands-off mode executes user-written script(s) with CNVpytor commands. Such scripts can be used as part of the processing pipeline where, for example, images of signals around called CNVs are generated and stored for possible future inspection. Through the viewer interface, it is possible to directly access Python and run code. This allows user to access some standard features of underlying libraries, e.g., matplotlib library can be used to customize plots.

CNVpytor can be used as the Python module. All functionalities, like reading and editing CNVpytor data files, and all calculation steps and visualizations can be performed by calling functions or classes. This way CNVpytor can be easily integrated in different platforms and computing environments; for example, CNVpytor can be run from Jupyter Notebook on a local machine or in cloud services, e.g., Google Colab. CNVpytor is also integrated into OmniTier's "Compstor Novos" variant calling workflow [29].

Figure 4. Novel features of visualization using data for HepG2 cell line. Genome-wide visualizations can be useful in some cases, like cancer studies or clinical applications like screening for chromosomal abnormalities. They also can be useful for quick insight in sequencing or single-cell amplification quality. To demonstrate genome-wide plot types, HapG2 immortal cell line sample is used. **A)** Manhattan and **B)** circular style plot of RD signal. Large CNVs and chromosome copy number changes are apparent. One can also judge about dispersion of the RD signal. The inner circle in B shows RD signal while outer shows minor allele frequency (MAF). Regions where we can see loss of MAF signal with normal RD, e.g., chromosome 14 or 22, are chromosomal CNN-LOH. **C)** Examples of smaller CNVs not apparent in the global view. Each CNV is about 15 Mbp. The displayed regions with multiple CNVs (that are possibly complex events) were hardly visible on the circular plot.

Data visualization and result curation

Visualization of multiple tracks/signals can be done interactively by mouse and by typing relevant commands as well as by running scripts with CNVpytor commands provided as inputs to CNVpytor. CNVpytor

has extended visualization capabilities with multiple novel features as compared to CNVnator. For each sample (i.e., input file) multiple data tracks such as RD signal, BAF of SNPs, and binned BAF likelihood can be displayed in an adjustable grid layout as specified by a user. Specifically, multiple regions across multiple samples can be plotted in parallel, facilitating comparison across samples and different genomic loci (**Fig. 2**). To get a global view, a user can visualize an entire genome in a linear or circular fashion (**Fig. 4, S1, S2**). Such a view can be useful in judging the quality of samples and in visually checking for aneuploidies.

Some additional features include GC-bias curve plot and 2D histogram (**Fig. 1D**), allele frequency distribution per region (**Fig. 1C**), and comparing RD distributions between two regions (**Fig. S3**). Figure resolution, layout grid, colors, marker size, titles, and plotting style are adjustable by the user. Overall CNVpytor has more functionalities than other available software (**Table S2**).

<u>Integration with JBrowse</u>

CNVpytor also has implemented functionality to export data into formats that can be embedded into JBrowse, a web-based genome browser used to visualize multiple related data tracks (**Fig. S4**). The export enables users to utilize JBrowse capabilities to visualize, compare, and cross-reference CNV calls with other data types (such as CHiP-seq, RNA-seq, ATAC-seq, etc.) and annotations across genome. Exported data provide 3 resolutions of RD and BAF tracks (1 kbp, 10 kbp, and 100 kbp bins) while the appropriate resolution is chosen automatically by JBrowse depending on the size of the visualized genomic region. Multiple .pytor files can be exported at once.

Alternatively, a user can utilize a lightweight CNVpytor plugin for JBrowse. The plugin takes information about coverage from a relatively small (as compared to BAM) VCF file and on the fly performs the read depth and BAF estimation, segmentation, and calling. For read depth analysis, the plugin fetches the information from the DP field in the VCF field and uses it as a proxy for actual coverage. Since for large bin sizes such an estimate corresponds well to the actual value (**Fig. S5**), the plugin enables quick and easy review of large copy number changes in a genome. For BAF analysis, the plugin conducts analyses the same way as a stand-alone application. All temporary values are stored in the browser cache for fast and interactive visualization of a genomic segment. As well as improving responsiveness by eliminating the network lag of a client-server application, this ensures that no information about a personal genome is transferred to external

servers. Once the analyses are complete, the results are instantly visualized using JBrowse's native capabilities. Usage cases of the plugin are: 1) quick and visual cross-referencing of copy number profiles between multiple samples and in relation to other data types, and 2) a review of a personal genome(s) for large CNVs in a simple user-friendly environment.

Conclusion

Development of new, maintenance, and improvement of existing bioinformatics tools are driven by changing data types, demands for newer and user-verifiable analyses, necessity for processing larger datasets, and the evolving nature of computational infrastructures and platforms. CNVpytor brings the functionality of its predecessor CNVnator to a new level and significantly expands it. CNVpytor is faster and virtually effortless to install, requires minimum space for storage, enables analysis of BAF for call confirmation and genotyping, provides users with instant and extended visualization and convenient functionality for result curation (including merging over multiple samples), and is equipped for integration with other tools. The utilized method is suitable to segment RD signal in the case of mosaic or somatic cancer samples CNAs, and the alteration will be called if its cell frequency is larger than 50% (**Fig. S6**). More accurate approach to discover cancer and mosaic CNAs is under development. The prototype of somatic CNA caller is functional, available in current version and documented on CNVpytor GitHub page. Provided good genome amplification and high sequencing coverage in a single cell, detection of CNAs in the cell becomes like detecting germline variation, i.e., every somatic variant will be present in one out of two haplotypes. Advancement of single cell amplification approaches [30] renders CNVpytor applicable to analysis of single cell data. Lightweight plugin for JBrowse enables on-the-fly visualization and analysis convenient for wide categories of users. Overall, CNVpytor establishes a framework for discovering and analyzing copy number changes from whole genome sequencing data either by an individual researcher or clinician or in a collaborative and shared environment.

Materials and Methods

RD analysis

Calculations for RD binning, mean-shift algorithm, partitioning and calling CNVs are explained in details in CNVnator paper [1]. The only difference in CNVpytor implementation is how information about GC content is obtained. For two versions GRCh37 (hg19) and GRCh38 of the human reference genome information about GC and AT content for each 100 base pair bins is provided as resource data within CNVpytor package. This way user does not need to have reference genome FASTA for GC correction. In spite of slightly different implementation called regions by CNVnator and CNVpytor overlap by over 99%. Additionally, CNVpytor can calculate read depth from coverage of imported variants (**Fig. 3**). This is an approximate but rather precise solution to cases when alignment file is not available (**Fig. S5**).

Variant data

CNVpytor imports information about SNPs and single letter indels form variant (VCF) file. All other variants are ignored. For each variant following data is stored in CNVpytor file: chromosome, position, reference base, alternative base, reference count (ref_i), alternative count (alt_i), quality, and genotype (0/1 or 1/1).

In the normal case with one copy of each haplotype, heterozygous SNPs are visible in half of the reads coming from that haplotype. In other words, B-allele frequency distributes around 1/2. Contrarily, in the regions with constitutional duplication of one haplotype, heterozygous SNPs are expected with frequency equals 2/3 or 1/3 depending are they located on duplicated haplotype or not. This split from value 1/2 can be visible in plot of BAFs vs position of variants as shown in the right panel of Figure 1. Similarly, for homozygous deletion complete loss of heterozygous SNPs is expected. In the case of somatic sub clonal CNA (e.g., frequently observed in cancer genomes), the ratio between haplotypes can be an arbitrary number depending on cell frequencies with the CNA. Consequently, the split in BAF plot varies from 0 through 1. Measuring the level of this split can provide useful information about type of CNV. Moreover, copy number neutral loss of heterozygosity (CNN-LOH) can be detected this way.

For each stored variant we can calculate two frequencies defined in following way:

- B-allele frequency (BAF): $BAF = \frac{alt_i}{ref_i + alt_i}$
- Minor allele frequency: $MAF = \min\left(\frac{alt_i}{ref_i + alt_i}, \frac{ref_i}{ref_i + alt_i}\right) = \min(BAF, 1 - BAF)$

One of next generation sequencing features is that some bases are not accessible for variant discovery using short reads, due to repetitive nature of the human genome. In the 1000 Genomes Project, genome mask is created to tabulate bases for variant discovery. There are about 74% of bases marked passed (P), what correspond to about 77% of non-N bases (1000 Genomes Project Consortium, 2015). We use that mask to filter out variants called in non-P regions. This way we eliminate around 22% of variants but there is benefit because with less false-positive heterozygous SNPs statistics is improved (**Fig. 1C**) and this improves quality of further calculations. The same way as GC content, information about strict mask P regions for two versions of human reference genome are stored in resource files that are part of CNVpytor package.

Calculating BAF likelihood function

The ratio between reads coming from one or another haplotype is distributed following binomial distribution. If counts are known then one can calculate likelihood function for that ratio:

$$L(p_i|alt_i, ref_i) = B(x; alt_i + 1, ref_i + 1) = \frac{1}{B(alt_i, ref_i)} {p_i}^{alt_i} (1 - p_i)^{ref_i},$$

where p_i is allele frequency for variant i and $B(alt_i, ref_i)$ is normalization constant.

By multiplying likelihood functions of individual variants in a bin one can obtain likelihood for each bin. This is true only if real value of that ratio does not change within a that bin. However, we are using non-phased variant counts, what means that there is 50% of chance that variant is coming from one or another haplotype. Frequency of a fixed haplotype is sometimes described by either BAF and 1 - BAF distributions.

In that case we have to use symmetrized beta function for likelihood:

$$L(p_i|alt_i, ref_i) = \frac{B(x; alt_i + 1, ref_i + 1) + B(x; ref_i + 1, alt_i + 1)}{2} = \frac{{p_i}^{alt_i}(1 - p_i)^{ref_i} + {p_i}^{ref_i}(1 - p_i)^{alt_i}}{2B(alt_i, ref_i)}$$

Likelihood function for each bin is calculated as a product of individual likelihood functions of variants within that bin:

$$L(p_b) \sim \prod_{i \in bin(b)} L(p_i | alt_i, ref_i) \sim \prod_i (p_i{}^{alt_i}(1-p_i)^{ref_i} + p_i{}^{ref_i}(1-p_i)^{alt_i}),$$

where p_b is allele frequency for bin b. To calculate likelihood functions we use discretization. Interval [0,0.5] is discretized using some resolution (default is 101 point) and for each point function is calculated by multiplying values of symmetrized beta functions for each variant. Position of maximum likelihood represents most probable BAF value in particular bin. Along with likelihood function average values of variant BAF and MAF are calculated per bin and stored in CNVpytor file together with counts of homozygous and heterozygous variants.

Filtering CNV calls

For each CNV call following values are calculated: (1) event type: "deletion" or "duplication"; (2) coordinates in the reference genome; (3) CNV size; (4) RD normalized to 1; (5) e-val1 – p value calculated using t-test statistics between RD difference in the region and global (i.e. across whole genome) mean; (6) e-val2 – p value from the probability of RD values within the region to be in the tails of a gaussian distribution of binned RD; (7) e-val3 – same as e-val1 but without first and last bin; (8) e-val4 – same as e-val2 but without first and last bin; (9) q0 – fraction of reads mapped with zero quality within call region; (10) pN – fraction of N bases (i.e., unassembled reference genome) within call region; (11) dG – distance to nearest gap in reference genome.

There are five parameters in viewer mode used for filtering calls: CNV size, e-val1, q0, pN and dG. Those parameters will define which calls CNVpytor will plot or print out. When calls are printed or exported CNVpytor optionally can generate graphical file(s) with plot of CNV call region containing user specified tracks.

Annotating CNV calls

To annotate called regions we use Ensembl REST API (overlap/region resource). It is an optional step requires web connection and is executed when calls are previewed by user or exported to an output file. The annotation is added in an additional column in the output and contains string with gene names, Ensembl

gene IDs and with information about position of genes relative to CNV (i.e., inside, covering, or intersecting left/right breakpoints of CNV region).

Genotyping

Copy number of a provided genomic region is calculated as a mean RD within the region divided by mean autosomal RD scaled by 2. To achieve better precision, first and last bin content are weighted by the fraction of overlap with the provided region. Optionally CNVpytor can provide additional values: (1) e-value from the probability of RD values within the region to be in the tails of a gaussian distribution of binned RD (analogous to e-val2); (2) q0 – fraction of reads mapped with q0 quality within call region; (3) pN – fraction of reference genome gaps (Ns) within call region; (4) BAF level estimated using maximum likelihood method; (5) number of homozygous variants within the region; (6) number of heterozygous variants within the region; (7) p-value based on BAF signal.

Merging calls over multiple regions

To make joint call set for multiple samples CNVpytor procceds in the following way:

1. Filter calls using user defined ranges for size, p-val, q0, pN and dG;
2. Sort all calls for all samples by start coordinate;
3. Select first call in that list that is not already processed and select calls from other samples with the reciprocal overlap larger than 50%;
4. For selected calls, calculate genotypes within the region of intersection and, optionally, annotate with overlapping genes.

If specified, for each joint CNV call CNVpytor will create graphical file with plot of call region containing user specified tracks.

Data format and compression

For data storage and compression, we used HDF5 file format and h5py python library. Additional compression is obtained by storing RD signal using 100 base pair bins. The same bin size is used for storing reference genome AT, GC and N content. Data organization within .pytor file is implemented in IO module

which can be used to open and read different datasets from an external application. Python library xlwt is used to generate spreadsheet files compatible with Microsoft Excel.

Visualizations

Matplotlib [31] python library is used for creating and storing visualizations. Different plotting styles available within matplotlib. Derived installed libraries can be used as well as variety of file formats for storing graphical data.

Module dependences

CNVpytor depends on several widely used python packages: requests 2.0 or higher, gnureadline, pathlib 1.0 or higher, pysam 0.15 or higher, numpy 1.16 or higher, scipy 1.1 or higher, matplotlib 2.2 or higher, h5py 2.9 or higher, xlwt 1.3 or higher. All dependences are available through pip installer that makes installation of CNVpytor straightforward.

Supplementary Information

Additional file: Figs. S1-S5

Acknowledgements

We thank Abyzov lab members for useful discussions and suggestions. We are grateful to users on the GitHub for useful suggestions and bug reports.

Authors' contributions

A.A. conceived and supervised this study. M.S. designed and developed CNVpytor software. A.P. and M.S. tested the software. A.P. and C.D developed plugin for JBrowse. I.H. co-supervised the development of plugin for JBrowse. M.S., A.P. and A.A. wrote the manuscript. All authors read and approved the final manuscript.

Funding

This study is supported by NCI grant U24CA220242 and funds from the Center for Individualized Medicine at Mayo Clinic.

Availability of Supporting Source Code and Requirements

Project name: CNVpytor

Project home page: https://github.com/abyzovlab/CNVpytor

Operating system(s): Platform independent

Programming language: Python

Other requirements: requests>=2.0, gnureadline, pathlib>=1.0, pysam>=0.15, numpy>=1.16, scipy>=1.1, matplotlib>=2.2, h5py>=2.9, xlsxwriter>=1.3, pathlib>=1.0

License: MIT License

RRID: SCR_021627

bio.tools ID: cnvpytor

Data availability

An archival copy of the code and links to data utilized to create figures are available via the GigaScience database, GigaDB [32].

Declarations

Ethics approval and consent to participate

Not applicable.

Consent for publication

Not applicable.

Competing interests

The authors declare that they have no competing interests.

References

1. Abyzov A, Urban AE, Snyder M and Gerstein M. CNVnator: an approach to discover, genotype, and characterize typical and atypical CNVs from family and population genome sequencing. Genome Res. 2011;21 6:974-84. doi:10.1101/gr.114876.110.
2. Mills RE, Walter K, Stewart C, Handsaker RE, Chen K, Alkan C, et al. Mapping copy number variation by population-scale genome sequencing. Nature. 2011;470 7332:59-65. doi:10.1038/nature09708.
3. Duan J, Zhang JG, Deng HW and Wang YP. Comparative studies of copy number variation detection methods for next-generation sequencing technologies. PLoS One. 2013;8 3:e59128. doi:10.1371/journal.pone.0059128.
4. Legault MA, Girard S, Lemieux Perreault LP, Rouleau GA and Dubé MP. Comparison of sequencing based CNV discovery methods using monozygotic twin quartets. PLoS One. 2015;10 3:e0122287. doi:10.1371/journal.pone.0122287.
5. Trost B, Walker S, Wang Z, Thiruvahindrapuram B, MacDonald JR, Sung WWL, et al. A Comprehensive Workflow for Read Depth-Based Identification of Copy-Number Variation from Whole-Genome Sequence Data. Am J Hum Genet. 2018;102 1:142-55. doi:10.1016/j.ajhg.2017.12.007.
6. Coll F, Preston M, Guerra-Assunção JA, Hill-Cawthorn G, Harris D, Perdigão J, et al. PolyTB: a genomic variation map for Mycobacterium tuberculosis. Tuberculosis (Edinb). 2014;94 3:346-54. doi:10.1016/j.tube.2014.02.005.
7. Cabañes FJ, Sanseverino W, Castellá G, Bragulat MR, Cigliano RA and Sánchez A. Rapid genome resequencing of an atoxigenic strain of Aspergillus carbonarius. Sci Rep. 2015;5:9086. doi:10.1038/srep09086.
8. Fuentes RR, Chebotarov D, Duitama J, Smith S, De la Hoz JF, Mohiyuddin M, et al. Structural variants in 3000 rice genomes. Genome Res. 2019;29 5:870-80. doi:10.1101/gr.241240.118.
9. Gordon SP, Priest H, Des Marais DL, Schackwitz W, Figueroa M, Martin J, et al. Genome diversity in Brachypodium distachyon: deep sequencing of highly diverse inbred lines. Plant J. 2014;79 3:361-74. doi:10.1111/tpj.12569.

10. Wallace JG, Bradbury PJ, Zhang N, Gibon Y, Stitt M and Buckler ES. Association mapping across numerous traits reveals patterns of functional variation in maize. PLoS Genet. 2014;10 12:e1004845. doi:10.1371/journal.pgen.1004845.

11. Choi JY, Bubnell JE and Aquadro CF. Population Genomics of Infectious and Integrated Wolbachia pipientis Genomes in Drosophila ananassae. Genome Biol Evol. 2015;7 8:2362-82. doi:10.1093/gbe/evv158.

12. Chain FJ, Feulner PG, Panchal M, Eizaguirre C, Samonte IE, Kalbe M, et al. Extensive copy-number variation of young genes across stickleback populations. PLoS Genet. 2014;10 12:e1004830. doi:10.1371/journal.pgen.1004830.

13. Yi G, Qu L, Liu J, Yan Y, Xu G and Yang N. Genome-wide patterns of copy number variation in the diversified chicken genomes using next-generation sequencing. BMC Genomics. 2014;15 1:962. doi:10.1186/1471-2164-15-962.

14. Hermsen R, de Ligt J, Spee W, Blokzijl F, Schäfer S, Adami E, et al. Genomic landscape of rat strain and substrain variation. BMC Genomics. 2015;16 1:357. doi:10.1186/s12864-015-1594-1.

15. Wang H, Wang C, Yang K, Liu J, Zhang Y, Wang Y, et al. Genome Wide Distributions and Functional Characterization of Copy Number Variations between Chinese and Western Pigs. PLoS One. 2015;10 7:e0131522. doi:10.1371/journal.pone.0131522.

16. Gokcumen O, Tischler V, Tica J, Zhu Q, Iskow RC, Lee E, et al. Primate genome architecture influences structural variation mechanisms and functional consequences. Proc Natl Acad Sci U S A. 2013;110 39:15764-9. doi:10.1073/pnas.1305904110.

17. Pezer Ž, Harr B, Teschke M, Babiker H and Tautz D. Divergence patterns of genic copy number variation in natural populations of the house mouse (Mus musculus domesticus) reveal three conserved genes with major population-specific expansions. Genome Res. 2015;25 8:1114-24. doi:10.1101/gr.187187.114.

18. Abel HJ, Larson DE, Regier AA, Chiang C, Das I, Kanchi KL, et al. Mapping and characterization of structural variation in 17,795 human genomes. Nature. 2020;583 7814:83-9. doi:10.1038/s41586-020-2371-0.

19. Sudmant PH, Rausch T, Gardner EJ, Handsaker RE, Abyzov A, Huddleston J, et al. An integrated map of structural variation in 2,504 human genomes. Nature. 2015;526 7571:75-81. doi:10.1038/nature15394.

20. Nagasaki M, Yasuda J, Katsuoka F, Nariai N, Kojima K, Kawai Y, et al. Rare variant discovery by deep whole-genome sequencing of 1,070 Japanese individuals. Nat Commun. 2015;6:8018. doi:10.1038/ncomms9018.

21. Han L, Zhao X, Benton ML, Perumal T, Collins RL, Hoffman GE, et al. Functional annotation of rare structural variation in the human brain. Nat Commun. 2020;11 1:2990. doi:10.1038/s41467-020-16736-1.

22. Guo H, Duyzend MH, Coe BP, Baker C, Hoekzema K, Gerdts J, et al. Genome sequencing identifies multiple deleterious variants in autism patients with more severe phenotypes. Genet Med. 2019;21 7:1611-20. doi:10.1038/s41436-018-0380-2.

23. Gilman P, Janzou S, Guittet D, Freeman J, DiOrio N, Blair N, et al. *PySAM (Python Wrapper for System Advisor Model" SAM")*. 2019. National Renewable Energy Lab.(NREL), Golden, CO (United States).

24. Peiffer DA, Le JM, Steemers FJ, Chang W, Jenniges T, Garcia F, et al. High-resolution genomic profiling of chromosomal aberrations using Infinium whole-genome genotyping. Genome Res. 2006;16 9:1136-48. doi:10.1101/gr.5402306.

25. Loh PR, Genovese G, Handsaker RE, Finucane HK, Reshef YA, Palamara PF, et al. Insights into clonal haematopoiesis from 8,342 mosaic chromosomal alterations. Nature. 2018;559 7714:350-5. doi:10.1038/s41586-018-0321-x.

26. Boeva V, Popova T, Bleakley K, Chiche P, Cappo J, Schleiermacher G, et al. Control-FREEC: a tool for assessing copy number and allelic content using next-generation sequencing data. Bioinformatics. 2012;28 3:423-5.

27. Zhu M, Need AC, Han Y, Ge D, Maia JM, Zhu Q, et al. Using ERDS to infer copy-number variants in high-coverage genomes. The American Journal of Human Genetics. 2012;91 3:408-21.

28. Auton A, Brooks LD, Durbin RM, Garrison EP, Kang HM, Korbel JO, et al. A global reference for human genetic variation. Nature. 2015;526 7571:68-74. doi:10.1038/nature15393.

29. OmniTier: https://www.omnitier.com/compstor-novos.

30. Gonzalez-Pena V, Natarajan S, Xia Y, Klein D, Carter R, Pang Y, et al. Accurate genomic variant detection in single cells with primary template-directed amplification. Proceedings of the National Academy of Sciences. 2021;118 24.

31. Hunter JD. Matplotlib: A 2D graphics environment. Computing in science & engineering. 2007;9 3:90-5.

32. Suvakov M, Panda A, Diesh C, Holmes I and Abyzov A. Supporting data for "CNVpytor: a tool for CNV detection and analysis from read depth and allele imbalance in whole genome sequencing" GigaScience Database. 2021; doi:http://doi.org/10.5524/100938.

Manuscript with figures (single spacing)

Click here to access/download;Manuscript;CNVpytor_manuscript_with_figur

CNVpytor: a tool for CNV detection and analysis from read depth and allele imbalance in whole genome sequencing

Milovan Suvakov[1,2], Arijit Panda[1,2], Colin Diesh[3], Ian Holmes[3], Alexej Abyzov[1,2,*]

[1]Department of Quantitative Health Sciences, Mayo Clinic, Rochester, MN

[2]Center for Individualized Medicine, Mayo Clinic, Rochester, MN

[3]Department of Bioengineering, University of California, Berkeley, USA

*Correspond author. Email: abyzov.alexej@mayo.edu

Keywords: copy number variations, copy number alternations, whole genome sequencing, Python

Abstract

Background: Detecting copy number variations (CNVs) and copy number alterations (CNAs) based on whole genome sequencing data is important for personalized genomics and treatment. CNVnator is one of the most popular tools for CNV/CNA discovery and analysis based on read depth (RD).

Findings: Herein, we present an extension of CNVnator developed in Python -- CNVpytor. CNVpytor inherits the reimplemented core engine of its predecessor and extends visualization, modularization, performance, and functionality. Additionally, CNVpytor uses B-allele frequency (BAF) likelihood information from single nucleotide polymorphism and small indels data as additional evidence for CNVs/CNAs and as primary information for copy number neutral losses of heterozygosity.

Conclusions: CNVpytor is significantly faster than CNVnator—particularly for parsing alignment files (2 to 20 times faster)—and has (20-50 times) smaller intermediate files. CNV calls can be filtered using several criteria, annotated, and merged over multiple samples. Modular architecture allows it to be used in shared and cloud environments such as Google Colab and Jupyter notebook. Data can be exported into JBrowse, while a lightweight plugin version of CNVpytor for JBrowse enables nearly instant and GUI-assisted analysis of CNVs by any user. CNVpytor release and the source code are available on GitHub at https://github.com/abyzovlab/CNVpytor under the MIT license.

Introduction

The continuous reduction of cost made the whole genome sequencing (WGS) to be widely used in different research projects and clinical applications. Consequently, many approaches for processing, analyzing, and visualizing WGS data have been developed and are being improved. Detection and analysis of copy number variations (CNVs) based on WGS data is one of them. Research directions related to the cancer genomics, single-cell sequencing, and somatic mosaicism create huge amounts of data and demands for processing on the cloud that require further improvements of CNV callers, moving to parallel processing, better compression, modular architecture, and new statistical methods.

CNVnator is a method for CNV analysis based on read depth (RD) of aligned reads. It was determined to have high sensitivity (86%-96%), low false-discovery rate (3%-20%), and high genotyping accuracy (93%-95%) for germline CNVs in a wide range of sizes from a few hundred base pairs to chromosome size events [1-5]. Since its development a decade ago, the tool has been widely used in different scientific areas by researchers around the world for detection of CNVs in a variety of species with different genome sizes: bacteria [6], fungi [7], plants [8-10], insects [11], fish [12], birds [13], mammals [14-17] and humans [2, 18-20]. It has been used to discover somatic variations in cancer and disease studies [21] and to find mosaic variants in human cells [22]. Although CNVnator was developed to detect germline CNVs, it is well-suited to discover copy number alteration (CNAs) present in a relatively high (>50%) fraction of cells, such as somatic alteration found in cancers. It was not, however, designed for nor capable of aiding analysis of copy number neutral changes.

Here we describe CNVpytor, a Python extension of CNVnator. CNVpytor inherits the reimplemented core engine of CNVnator and extends visualization, modularization, performance, and functionality. Along with RD data, it enables consideration of allele frequency of single nucleotide polymorphism (SNP) and small indels as an additional source of information for the analysis of CNV/CNA and copy number neutral variations. Along with RD data, this information can be used for genotyping genomic regions and visualization.

Results

Analysis of RD signal

CNVpytor inherits the RD analysis approach developed in CNVnator [1]. Briefly, it consists of the following steps: reading alignment file and extracting RD signal, binning RD signal, correcting the signal for GC bias, segmenting the signal using the mean-shift technique, and calling CNVs (**Fig. 1**). RD signal can be parsed from BAM, SAM, or CRAM alignment files and is counted in 100 bp intervals, resulting in a small footprint of intermediate .pytor files in HDF5 format (**Table 1**). Because of using the pysam [23] library for parsing, this step (the most time-consuming one) is parallelized and can be conducted very efficiently, particularly in comparison with the older tool (**Table 1**). The binning step integrates RD over larger bins that are limited to multiples of initially stored 100 bp bins. Bin size can be adjusted by user depending on application. Depending on sequence coverage recommended minimal bin size is provided in Supplement (**Table S1**). Next, technical biases in the read depth signal that are correlated with GC content (so-called GC biases) are removed using GC correction procedure. For the human reference genomes GRCh37 and GRCh38 per bin, GC content is pre-calculated and supplied as a resource with the CNVpytor package. For other genomes, GC content can be calculated during runtime from a provided FASTA file or precalculated and added to the CNVpytor resource for future use. Once information about read coverage (and variants, see below) is extracted from an alignment file, the following analysis steps take place (i.e., read input and write output) with the same file. As a result, histograms for each processed bin size and information about CNV calls, including coordinates, different statistics, and p-values are all stored in one .pytor file and can be extracted into Excel (TSV file) or a VCF file.

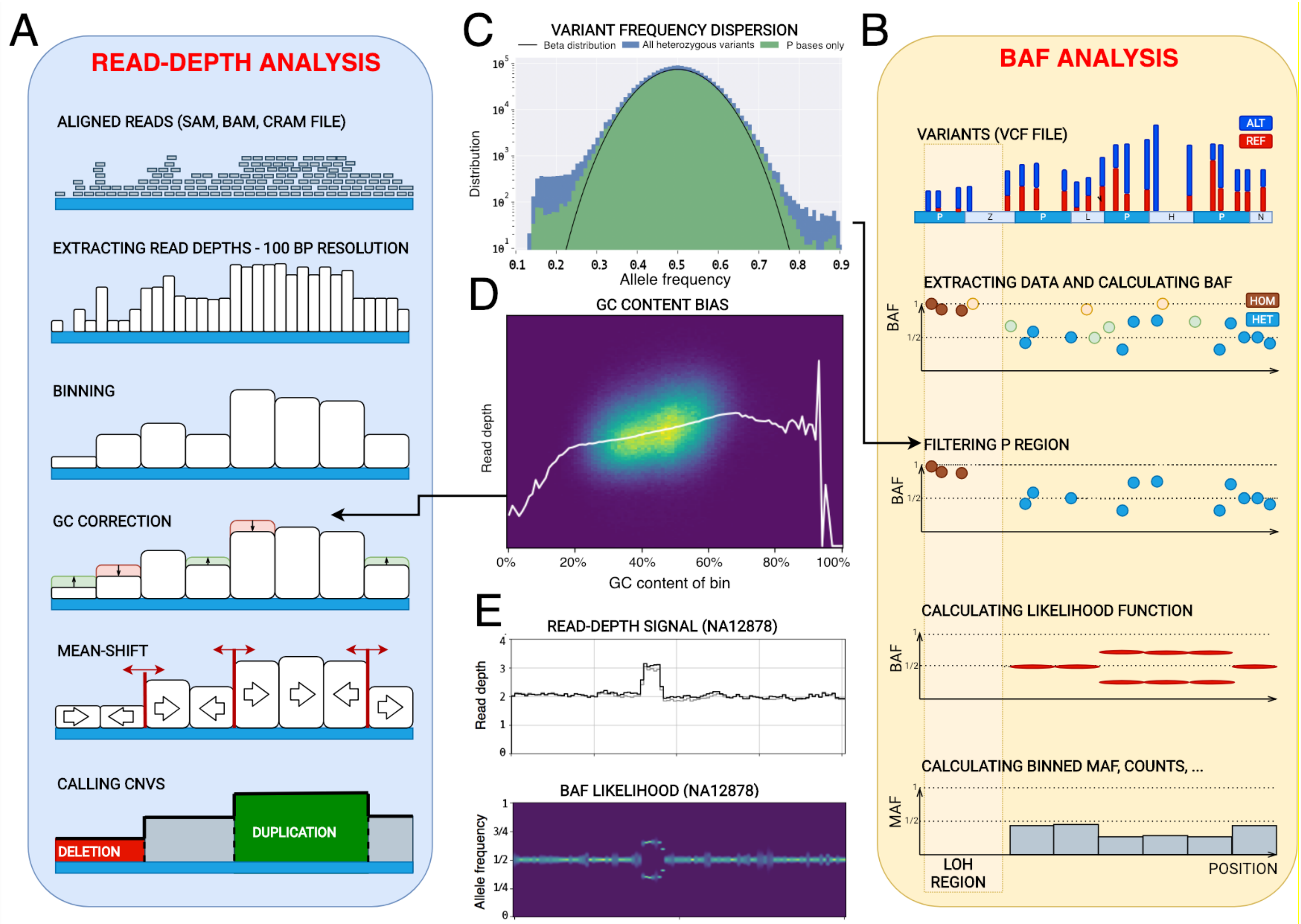

Figure 1. Schematics of core algorithm and data processing steps. (A) Read depth analysis steps include parsing alignment file, calculating and storing read depths in 100 bp intervals, binning using user-specified bin size, correcting RD for GC bias, segmenting by mean-shift, and calling CNVs. (B) B-allele frequency analysis steps include reading variant file storing the data about SNPs and small indels, filtering variants using strict mask, calculating BAF for heterozygous variants (HETs), and calculating likelihood function for bins. For CNVs, BAF signal splits away from value 0.5 expected for HETs. (C) Distribution of the variant allele frequency for all variants and variants within strict mask as defined by the 1000 Genomes Project. Black line shows fit by gaussian distribution. (D) An example of RD depending on GC within bin. Statistics of RD signal within bins of the same percentage of GC content is used to correct for GC bias in the signal. White line represents average RD level for bins with given GC content. (E) An example of RD and BAF signals for a germline duplication in NA12878 sample (raw RD signal is in grey, GC-corrected RD signal is in black, brighter color of BAF likelihood corresponds to higher values of the likelihood).

Table 1. Efficiency of parsing alignment file on modern computers in relation to sequencing coverage and engaged number of CPU cores.

**Sequencing coverage of the human genome**	**Parsing time**				**File size**			
		**CNVpytor**				**CNVpytor**		
	**CNVnator**	**4 cores**	**8 cores**	**23 cores**	**CNVnator**	**RD parsing**	**BAF parsing**	**With 1 & 10 kbp bins**
5X	10 min	5 min	3 min	<2 min	1 Gb	18 Mb	20 Mb	250 Mb
30X	1 h	28 min	18 min	10 min	1.5 Gb	19 Mb	20 Mb	250 Mb
100X	3.3 h	1.5 h	1 h	33 min	2 Gb	20 Mb	20 Mb	250 Mb

Analysis of variant data

A novel feature of CNVpytor is the analysis of information from SNPs and small indels imported from a VCF file. An imbalance in the number of haplotypes can be measured using allele frequencies traditionally referred to as B-allele frequency (BAF) [24-27]. The main advantage of using BAF compared to RD is that BAF values do not require normalization and are distributed around 0.5 by binomial distribution for heterozygous variants (HETs). Additionally, BAF is complementary to RD signal, as it changes for copy number neutral events such as loss of heterozygosity. However, BAF dispersion can be measured incorrectly due to systematic misalignment particularly in repeat regions, incomplete reference genome, or site-specific noise in sequencing data. To mitigate this issue, we filtered out HETs in the fraction of genome that is inaccessible to short read technologies, as defined by the strict mask from the 1000 Genomes Project [28]. Such filtering removes almost all HETs with outlier values of BAF, while values for the retained variants closely follow binomial distribution (**Fig. 1C**). To integrate BAF information within bins, CNVpytor calculates the likelihood function that describes an imbalance between haplotypes (see **Methods**). Currently, BAF information is used when genotyping a specific genomic region where, along with estimated copy number, the output contains the average BAF level and two independent p-values calculated from RD and BAF signal. Variant data can also be plotted in parallel with RD signal (**Fig. 2**). Same as for RD signal, binned information calculated from variants is stored in and can be extracted from .pytor file.

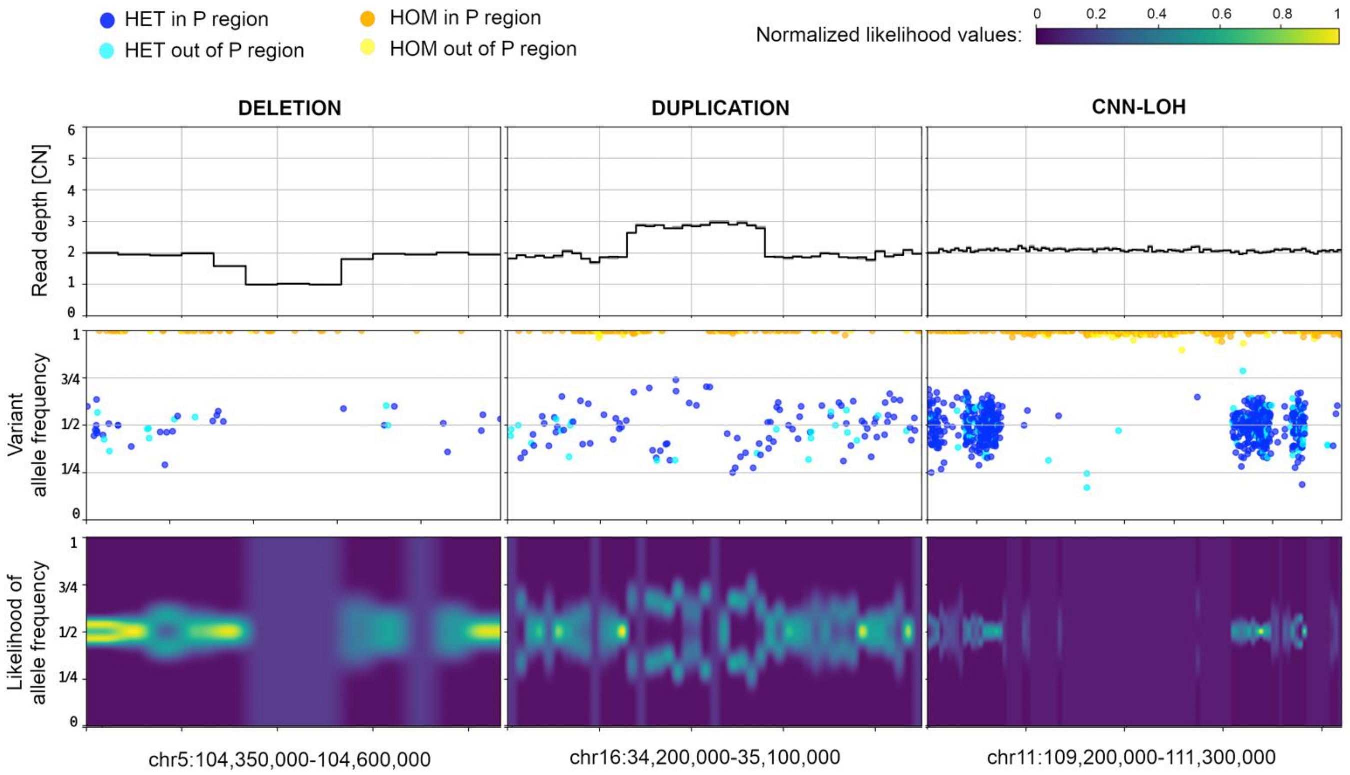

Figure 2. BAF signal corroborates and complements RD signal. Example of CNVpytor *region* plots produced for deletion (left), duplication (middle), and CNN-LOH (right) for NA12878 sample. Within the coordinates of heterozygous deletion, there is a 50% drop in RD signal and a loss in heterozygosity in BAF signal (i.e., no heterozygous SNPs in the region). Duplication of one haplotype results in the increase of RD signal by 50% and in a split in VAF distribution of SNPs and a split in BAF likelihood function. In the CNN-LOH region, few reliable heterozygous SNPs are detected while RD signal does not change. Likelihood function values are normalized to maximal across the range.

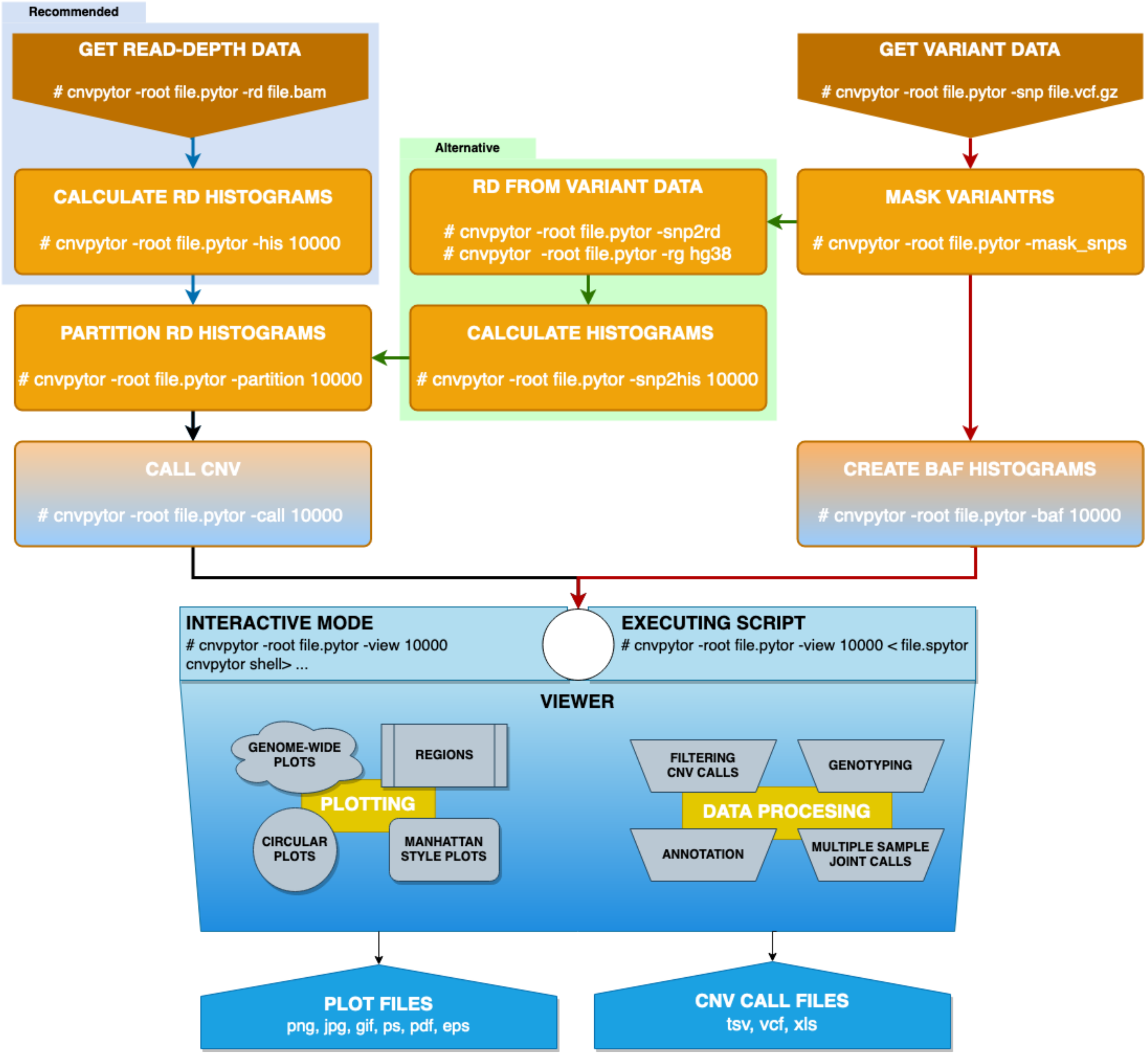

Figure 3. CNVpytor workflow. Steps used in data processing. On the left: reading RD data from alignment file, creating histograms, segmentation, calling CNVs. On the right: reading SNP and indel data from VCF file, filtering variants using strict mask, calculating histograms and likelihood function. In the middle: alternatively, if alignment file is not available RD signals can be calculated from variant data (green arrows). Visualization using both RD and/or BAF data can be done from an interactive command line interface or automatically by running script file.

<u>Running CNVpytor</u>

CNVpytor is to be run in a series of steps (**Fig. 3)**. For enhanced flexibility, RD and BAF processing workflows proceed in parallel. In this way each workflow can be run at different times or even on different computers. For example, data parsing steps can be run on a cloud where data (i.e., alignment files) is accessible, resulting in less than 25 Mb .pytor files that then can be copied to local computer/cluster where the remaining calculation steps will be performed. If necessary, a user can run additional calculations (e.g., conduct processing with different bin size) using the same .pytor file in the future, allowing for further flexibility in data analysis.

Routine processing steps can be followed by CNV visualization and analysis in the Viewer session, which can be interactive or hands-off. Implementation of interactive mode is inspired by a Linux shell with tab

completion and with a help page similar to the man pages. In this mode a user can instantly make various visualizations, preview and filter CNV calls, annotate calls, create joint calls across multiple samples, and genotype specified regions. The viewer does not save results into the .pytor file, and outputs are printed and plotted on the screen or exported to an output file(s). Hands-off mode executes user-written script(s) with CNVpytor commands. Such scripts can be used as part of the processing pipeline where, for example, images of signals around called CNVs are generated and stored for possible future inspection. Through the viewer interface, it is possible to directly access Python and run code. This allows user to access some standard features of underlying libraries, e.g., matplotlib library can be used to customize plots.

CNVpytor can be used as the Python module. All functionalities, like reading and editing CNVpytor data files, and all calculation steps and visualizations can be performed by calling functions or classes. This way CNVpytor can be easily integrated in different platforms and computing environments; for example, CNVpytor can be run from Jupyter Notebook on a local machine or in cloud services, e.g., Google Colab. CNVpytor is also integrated into OmniTier's "Compstor Novos" variant calling workflow [29].

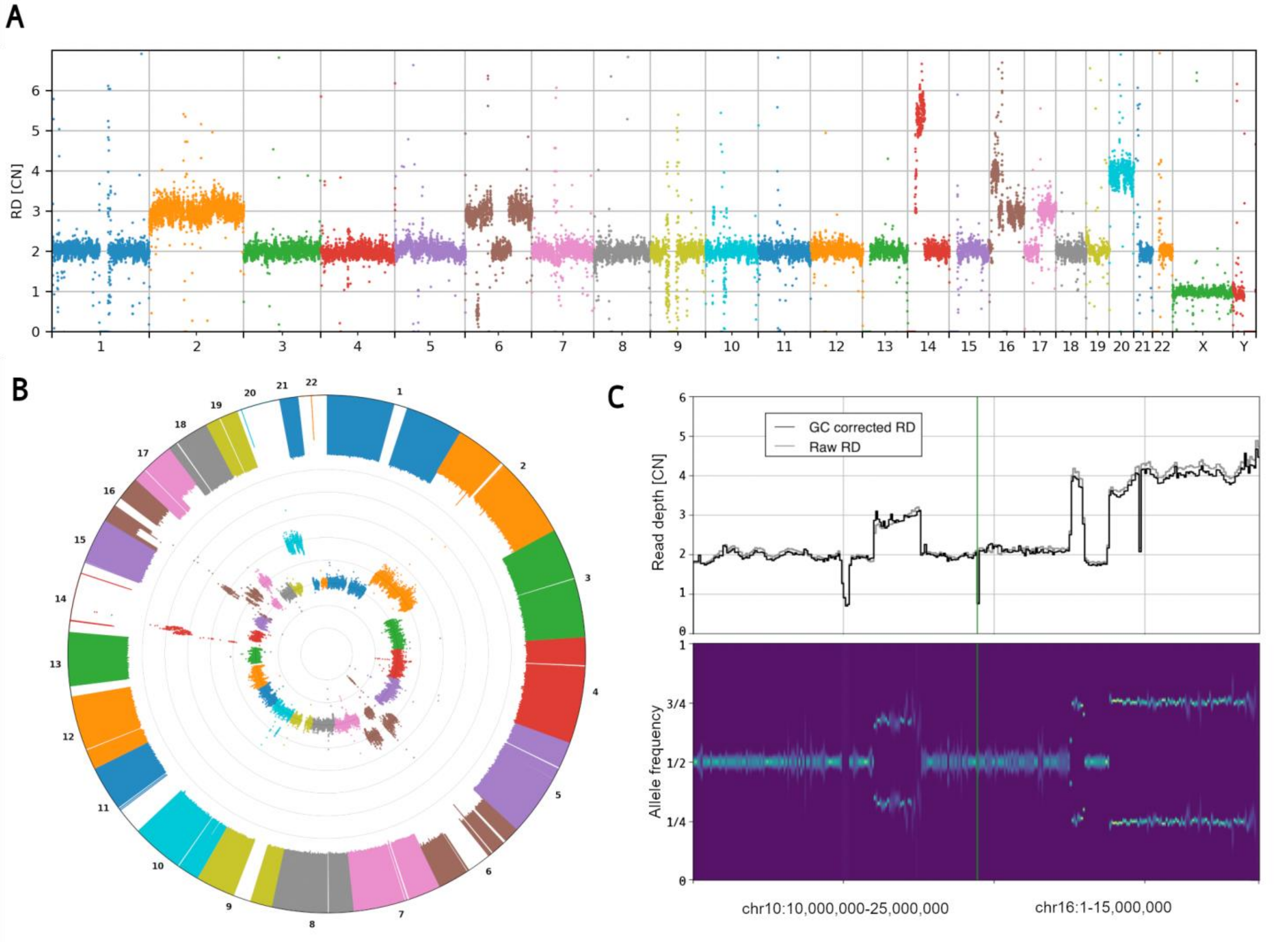

Figure 4. Novel features of visualization using data for HepG2 cell line. Genome-wide visualizations can be useful in some cases, like cancer studies or clinical applications like screening for chromosomal abnormalities. They also can be useful for quick insight in sequencing or single-cell amplification quality. To demonstrate genome-wide plot types, HapG2 immortal cell line sample is used. **A)** Manhattan and **B)** circular style plot of RD signal. Large CNVs and chromosome copy number changes are apparent. One can also judge about dispersion of the RD signal. The inner circle in B shows RD signal while outer shows minor allele frequency (MAF). Regions where we can see loss of MAF signal with normal RD, e.g., chromosome 14 or 22, are chromosomal CNN-LOH. **C)** Examples of smaller CNVs not apparent in the global view. Each CNV is about 15 Mbp. The displayed regions with multiple CNVs (that are possibly complex events) were hardly visible on the circular plot.

Data visualization and result curation

Visualization of multiple tracks/signals can be done interactively by mouse and by typing relevant commands as well as by running scripts with CNVpytor commands provided as inputs to CNVpytor. CNVpytor has extended visualization capabilities with multiple novel features as compared to CNVnator. For each sample (i.e., input file) multiple data tracks such as RD signal, BAF of SNPs, and binned BAF likelihood can be displayed in an adjustable grid layout as specified by a user. Specifically, multiple regions across multiple samples can be plotted in parallel, facilitating comparison across samples and different genomic loci (**Fig. 2**). To get a global view, a user can visualize an entire genome in a linear or circular fashion (**Fig. 4, S1, S2**). Such a view can be useful in judging the quality of samples and in visually checking for aneuploidies.

Some additional features include GC-bias curve plot and 2D histogram (**Fig. 1D**), allele frequency distribution per region (**Fig. 1C**), and comparing RD distributions between two regions (**Fig. S3**). Figure resolution, layout grid, colors, marker size, titles, and plotting style are adjustable by the user. Overall CNVpytor has more functionalities than other available software (**Table S2**).

Integration with JBrowse

CNVpytor also has implemented functionality to export data into formats that can be embedded into JBrowse, a web-based genome browser used to visualize multiple related data tracks (**Fig. S4**). The export enables users to utilize JBrowse capabilities to visualize, compare, and cross-reference CNV calls with other data types (such as CHiP-seq, RNA-seq, ATAC-seq, etc.) and annotations across genome. Exported data provide 3 resolutions of RD and BAF tracks (1 kbp, 10 kbp, and 100 kbp bins) while the appropriate resolution is chosen automatically by JBrowse depending on the size of the visualized genomic region. Multiple .pytor files can be exported at once.

Alternatively, a user can utilize a lightweight CNVpytor plugin for JBrowse. The plugin takes information about coverage from a relatively small (as compared to BAM) VCF file and on the fly performs the read depth and BAF estimation, segmentation, and calling. For read depth analysis, the plugin fetches the information from the DP field in the VCF field and uses it as a proxy for actual coverage. Since for large bin sizes such an estimate corresponds well to the actual value (**Fig. S5**), the plugin enables quick and easy review of large copy number changes in a genome. For BAF analysis, the plugin conducts analyses the same way as a stand-alone application. All temporary values are stored in the browser cache for fast and interactive visualization of a genomic segment. As well as improving responsiveness by eliminating the network lag of a client-server application, this ensures that no information about a personal genome is transferred to external servers. Once the analyses are complete, the results are instantly visualized using JBrowse's native capabilities. Usage cases of the plugin are: 1) quick and visual cross-referencing of copy number profiles between multiple samples and in relation to other data types, and 2) a review of a personal genome(s) for large CNVs in a simple user-friendly environment.

Conclusion

Development of new, maintenance, and improvement of existing bioinformatics tools are driven by changing data types, demands for newer and user-verifiable analyses, necessity for processing larger datasets, and the evolving nature of computational infrastructures and platforms. CNVpytor brings the functionality of its predecessor CNVnator to a new level and significantly expands it. CNVpytor is faster and virtually effortless to install, requires minimum space for storage, enables analysis of BAF for call confirmation and genotyping, provides users with instant and extended visualization and convenient functionality for result curation (including merging over multiple samples), and is equipped for integration with other tools. The utilized method is suitable to segment RD signal in the case of mosaic or somatic cancer samples CNAs, and the alteration will be called if its cell frequency is larger than 50% (**Fig. S6**). More accurate approach to discover cancer and mosaic CNAs is under development. The prototype of somatic CNA caller is functional, available in current version and documented on CNVpytor GitHub page. Provided good genome amplification and high sequencing coverage in a single cell, detection of CNAs in the cell becomes like detecting germline variation, i.e., every somatic variant will be present in one out of two haplotypes. Advancement of single cell amplification approaches [30] renders CNVpytor applicable to analysis of single cell data. Lightweight plugin for JBrowse enables on-the-fly visualization and analysis convenient for wide categories of users. Overall, CNVpytor establishes a framework for discovering and analyzing copy number changes from whole genome sequencing data either by an individual researcher or clinician or in a collaborative and shared environment.

Materials and Methods

RD analysis

Calculations for RD binning, mean-shift algorithm, partitioning and calling CNVs are explained in details in CNVnator paper [1]. The only difference in CNVpytor implementation is how information about GC content is obtained. For two versions GRCh37 (hg19) and GRCh38 of the human reference genome information about GC and AT content for each 100 base pair bins is provided as resource data within CNVpytor package. This way user does not need to have reference genome FASTA for GC correction. In spite of slightly different implementation called regions by CNVnator and CNVpytor overlap by over 99%. Additionally, CNVpytor can calculate read depth from coverage of imported variants (**Fig. 3**). This is an approximate but rather precise solution to cases when alignment file is not available (**Fig. S5**).

Variant data

CNVpytor imports information about SNPs and single letter indels form variant (VCF) file. All other variants are ignored. For each variant following data is stored in CNVpytor file: chromosome, position, reference base, alternative base, reference count (ref_i), alternative count (alt_i), quality, and genotype (0/1 or 1/1).

In the normal case with one copy of each haplotype, heterozygous SNPs are visible in half of the reads coming from that haplotype. In other words, B-allele frequency distributes around 1/2. Contrarily, in the regions with constitutional duplication of one haplotype, heterozygous SNPs are expected with frequency equals 2/3 or 1/3 depending are they located on duplicated haplotype or not. This split from value 1/2 can be visible in plot of BAFs vs position of variants as shown in the right panel of Figure 1. Similarly, for homozygous deletion complete loss of heterozygous SNPs is expected. In the case of somatic sub clonal CNA (e.g., frequently observed in cancer genomes), the ratio between haplotypes can be an arbitrary number depending on cell frequencies with the CNA. Consequently, the split in BAF plot varies from 0 through 1. Measuring the level of this split can provide useful information about type of CNV. Moreover, copy number neutral loss of heterozygosity (CNN-LOH) can be detected this way.

For each stored variant we can calculate two frequencies defined in following way:

- B-allele frequency (BAF): $BAF = \frac{alt_i}{ref_i + alt_i}$
- Minor allele frequency: $MAF = \min\left(\frac{alt_i}{ref_i + alt_i}, \frac{ref_i}{ref_i + alt_i}\right) = \min(BAF, 1 - BAF)$

One of next generation sequencing features is that some bases are not accessible for variant discovery using short reads, due to repetitive nature of the human genome. In the 1000 Genomes Project, genome mask is created to tabulate bases for variant discovery. There are about 74% of bases marked passed (P), what correspond to about 77% of non-N bases (1000 Genomes Project Consortium, 2015). We use that mask to filter out variants called in non-P regions. This way we eliminate around 22% of variants but there is benefit because with less false-positive heterozygous SNPs statistics is improved (**Fig. 1C**) and this improves quality of further calculations. The same way as GC content, information about strict mask P regions for two versions of human reference genome are stored in resource files that are part of CNVpytor package.

Calculating BAF likelihood function

The ratio between reads coming from one or another haplotype is distributed following binomial distribution. If counts are known then one can calculate likelihood function for that ratio:

$$L(p_i|alt_i, ref_i) = B(x; alt_i + 1, ref_i + 1) = \frac{1}{B(alt_i, ref_i)} p_i^{alt_i} (1 - p_i)^{ref_i},$$

where p_i is allele frequency for variant i and $B(alt_i, ref_i)$ is normalization constant.

By multiplying likelihood functions of individual variants in a bin one can obtain likelihood for each bin. This is true only if real value of that ratio does not change within a that bin. However, we are using non-phased variant counts, what means that there is 50% of chance that variant is coming from one or another haplotype. Frequency of a fixed haplotype is sometimes described by either BAF and 1 - BAF distributions.

In that case we have to use symmetrized beta function for likelihood:

$$L(p_i|alt_i, ref_i) = \frac{B(x; alt_i + 1, ref_i + 1) + B(x; ref_i + 1, alt_i + 1)}{2} = \frac{p_i^{alt_i}(1 - p_i)^{ref_i} + p_i^{ref_i}(1 - p_i)^{alt_i}}{2B(alt_i, ref_i)}$$

Likelihood function for each bin is calculated as a product of individual likelihood functions of variants within that bin:

$$L(p_b) \sim \prod_{i\in bin(b)} L(p_i|alt_i, ref_i) \sim \prod_i (p_i{}^{alt_i}(1-p_i)^{ref_i} + p_i{}^{ref_i}(1-p_i)^{alt_i}),$$

where p_b is allele frequency for bin b. To calculate likelihood functions we use discretization. Interval [0,0.5] is discretized using some resolution (default is 101 point) and for each point function is calculated by multiplying values of symmetrized beta functions for each variant. Position of maximum likelihood represents most probable BAF value in particular bin. Along with likelihood function average values of variant BAF and MAF are calculated per bin and stored in CNVpytor file together with counts of homozygous and heterozygous variants.

<u>Filtering CNV calls</u>

For each CNV call following values are calculated: (1) event type: "deletion" or "duplication"; (2) coordinates in the reference genome; (3) CNV size; (4) RD normalized to 1; (5) e-val1 – p value calculated using t-test statistics between RD difference in the region and global (i.e. across whole genome) mean; (6) e-val2 – p value from the probability of RD values within the region to be in the tails of a gaussian distribution of binned RD; (7) e-val3 – same as e-val1 but without first and last bin; (8) e-val4 – same as e-val2 but without first and last bin; (9) q0 – fraction of reads mapped with zero quality within call region; (10) pN – fraction of N bases (i.e., unassembled reference genome) within call region; (11) dG – distance to nearest gap in reference genome.

There are five parameters in viewer mode used for filtering calls: CNV size, e-val1, q0, pN and dG. Those parameters will define which calls CNVpytor will plot or print out. When calls are printed or exported CNVpytor optionally can generate graphical file(s) with plot of CNV call region containing user specified tracks.

<u>Annotating CNV calls</u>

To annotate called regions we use Ensembl REST API (overlap/region resource). It is an optional step requires web connection and is executed when calls are previewed by user or exported to an output file. The annotation is added in an additional column in the output and contains string with gene names, Ensembl gene IDs and with information about position of genes relative to CNV (i.e., inside, covering, or intersecting left/right breakpoints of CNV region).

<u>Genotyping</u>

Copy number of a provided genomic region is calculated as a mean RD within the region divided by mean autosomal RD scaled by 2. To achieve better precision, first and last bin content are weighted by the fraction of overlap with the provided region. Optionally CNVpytor can provide additional values: (1) e-value from the probability of RD values within the region to be in the tails of a gaussian distribution of binned RD (analogous to e-val2); (2) q0 – fraction of reads mapped with q0 quality within call region; (3) pN – fraction of reference genome gaps (Ns) within call region; (4) BAF level estimated using maximum likelihood method; (5) number of homozygous variants within the region; (6) number of heterozygous variants within the region; (7) p-value based on BAF signal.

<u>Merging calls over multiple regions</u>

To make joint call set for multiple samples CNVpytor procceds in the following way:

1. Filter calls using user defined ranges for size, p-val, q0, pN and dG;
2. Sort all calls for all samples by start coordinate;
3. Select first call in that list that is not already processed and select calls from other samples with the reciprocal overlap larger than 50%;
4. For selected calls, calculate genotypes within the region of intersection and, optionally, annotate with overlapping genes.

If specified, for each joint CNV call CNVpytor will create graphical file with plot of call region containing user specified tracks.

<u>Data format and compression</u>

For data storage and compression, we used HDF5 file format and h5py python library. Additional compression is obtained by storing RD signal using 100 base pair bins. The same bin size is used for storing reference genome AT, GC and N content. Data organization within .pytor file is implemented in IO module which can be used to open and read different datasets from an external application. Python library xlwt is used to generate spreadsheet files compatible with Microsoft Excel.

Visualizations

Matplotlib [31] python library is used for creating and storing visualizations. Different plotting styles available within matplotlib. Derived installed libraries can be used as well as variety of file formats for storing graphical data.

Module dependences

CNVpytor depends on several widely used python packages: requests 2.0 or higher, gnureadline, pathlib 1.0 or higher, pysam 0.15 or higher, numpy 1.16 or higher, scipy 1.1 or higher, matplotlib 2.2 or higher, h5py 2.9 or higher, xlwt 1.3 or higher. All dependences are available through pip installer that makes installation of CNVpytor straightforward.

Supplementary Information

Additional file: Figs. S1-S5

Acknowledgements

We thank Abyzov lab members for useful discussions and suggestions. We are grateful to users on the GitHub for useful suggestions and bug reports.

Authors' contributions

A.A. conceived and supervised this study. M.S. designed and developed CNVpytor software. A.P. and M.S. tested the software. A.P. and C.D developed plugin for JBrowse. I.H. co-supervised the development of plugin for JBrowse. M.S., A.P. and A.A. wrote the manuscript. All authors read and approved the final manuscript.

Funding

This study is supported by NCI grant U24CA220242 and funds from the Center for Individualized Medicine at Mayo Clinic.

Availability of Supporting Source Code and Requirements

Project name: CNVpytor
Project home page: https://github.com/abyzovlab/CNVpytor
Operating system(s): Platform independent
Programming language: Python
Other requirements: requests>=2.0, gnureadline, pathlib>=1.0, pysam>=0.15, numpy>=1.16, scipy>=1.1, matplotlib>=2.2, h5py>=2.9, xlsxwriter>=1.3, pathlib>=1.0
License: MIT License
RRID: SCR_021627
bio.tools ID: cnvpytor

Data availability

An archival copy of the code and links to data utilized to create figures are available via the GigaScience database, GigaDB [32].

Declarations

Ethics approval and consent to participate

Not applicable.

Consent for publication

Not applicable.

Competing interests

The authors declare that they have no competing interests.

References

1. Abyzov A, Urban AE, Snyder M and Gerstein M. CNVnator: an approach to discover, genotype, and characterize typical and atypical CNVs from family and population genome sequencing. Genome Res. 2011;21 6:974-84. doi:10.1101/gr.114876.110.
2. Mills RE, Walter K, Stewart C, Handsaker RE, Chen K, Alkan C, et al. Mapping copy number variation by population-scale genome sequencing. Nature. 2011;470 7332:59-65. doi:10.1038/nature09708.
3. Duan J, Zhang JG, Deng HW and Wang YP. Comparative studies of copy number variation detection methods for next-generation sequencing technologies. PLoS One. 2013;8 3:e59128. doi:10.1371/journal.pone.0059128.
4. Legault MA, Girard S, Lemieux Perreault LP, Rouleau GA and Dubé MP. Comparison of sequencing based CNV discovery methods using monozygotic twin quartets. PLoS One. 2015;10 3:e0122287. doi:10.1371/journal.pone.0122287.
5. Trost B, Walker S, Wang Z, Thiruvahindrapuram B, MacDonald JR, Sung WWL, et al. A Comprehensive Workflow for Read Depth-Based Identification of Copy-Number Variation from Whole-Genome Sequence Data. Am J Hum Genet. 2018;102 1:142-55. doi:10.1016/j.ajhg.2017.12.007.
6. Coll F, Preston M, Guerra-Assunção JA, Hill-Cawthorn G, Harris D, Perdigão J, et al. PolyTB: a genomic variation map for Mycobacterium tuberculosis. Tuberculosis (Edinb). 2014;94 3:346-54. doi:10.1016/j.tube.2014.02.005.
7. Cabañes FJ, Sanseverino W, Castellá G, Bragulat MR, Cigliano RA and Sánchez A. Rapid genome resequencing of an atoxigenic strain of Aspergillus carbonarius. Sci Rep. 2015;5:9086. doi:10.1038/srep09086.
8. Fuentes RR, Chebotarov D, Duitama J, Smith S, De la Hoz JF, Mohiyuddin M, et al. Structural variants in 3000 rice genomes. Genome Res. 2019;29 5:870-80. doi:10.1101/gr.241240.118.
9. Gordon SP, Priest H, Des Marais DL, Schackwitz W, Figueroa M, Martin J, et al. Genome diversity in Brachypodium distachyon: deep sequencing of highly diverse inbred lines. Plant J. 2014;79 3:361-74. doi:10.1111/tpj.12569.
10. Wallace JG, Bradbury PJ, Zhang N, Gibon Y, Stitt M and Buckler ES. Association mapping across numerous traits reveals patterns of functional variation in maize. PLoS Genet. 2014;10 12:e1004845. doi:10.1371/journal.pgen.1004845.
11. Choi JY, Bubnell JE and Aquadro CF. Population Genomics of Infectious and Integrated Wolbachia pipientis Genomes in Drosophila ananassae. Genome Biol Evol. 2015;7 8:2362-82. doi:10.1093/gbe/evv158.
12. Chain FJ, Feulner PG, Panchal M, Eizaguirre C, Samonte IE, Kalbe M, et al. Extensive copy-number variation of young genes across stickleback populations. PLoS Genet. 2014;10 12:e1004830. doi:10.1371/journal.pgen.1004830.
13. Yi G, Qu L, Liu J, Yan Y, Xu G and Yang N. Genome-wide patterns of copy number variation in the diversified chicken genomes using next-generation sequencing. BMC Genomics. 2014;15 1:962. doi:10.1186/1471-2164-15-962.
14. Hermsen R, de Ligt J, Spee W, Blokzijl F, Schäfer S, Adami E, et al. Genomic landscape of rat strain and substrain variation. BMC Genomics. 2015;16 1:357. doi:10.1186/s12864-015-1594-1.
15. Wang H, Wang C, Yang K, Liu J, Zhang Y, Wang Y, et al. Genome Wide Distributions and Functional Characterization of Copy Number Variations between Chinese and Western Pigs. PLoS One. 2015;10 7:e0131522. doi:10.1371/journal.pone.0131522.
16. Gokcumen O, Tischler V, Tica J, Zhu Q, Iskow RC, Lee E, et al. Primate genome architecture influences structural variation mechanisms and functional consequences. Proc Natl Acad Sci U S A. 2013;110 39:15764-9. doi:10.1073/pnas.1305904110.
17. Pezer Ž, Harr B, Teschke M, Babiker H and Tautz D. Divergence patterns of genic copy number variation in natural populations of the house mouse (Mus musculus domesticus) reveal three conserved genes with major population-specific expansions. Genome Res. 2015;25 8:1114-24. doi:10.1101/gr.187187.114.
18. Abel HJ, Larson DE, Regier AA, Chiang C, Das I, Kanchi KL, et al. Mapping and characterization of structural variation in 17,795 human genomes. Nature. 2020;583 7814:83-9. doi:10.1038/s41586-020-2371-0.
19. Sudmant PH, Rausch T, Gardner EJ, Handsaker RE, Abyzov A, Huddleston J, et al. An integrated map of structural variation in 2,504 human genomes. Nature. 2015;526 7571:75-81. doi:10.1038/nature15394.

20. Nagasaki M, Yasuda J, Katsuoka F, Nariai N, Kojima K, Kawai Y, et al. Rare variant discovery by deep whole-genome sequencing of 1,070 Japanese individuals. Nat Commun. 2015;6:8018. doi:10.1038/ncomms9018.
21. Han L, Zhao X, Benton ML, Perumal T, Collins RL, Hoffman GE, et al. Functional annotation of rare structural variation in the human brain. Nat Commun. 2020;11 1:2990. doi:10.1038/s41467-020-16736-1.
22. Guo H, Duyzend MH, Coe BP, Baker C, Hoekzema K, Gerdts J, et al. Genome sequencing identifies multiple deleterious variants in autism patients with more severe phenotypes. Genet Med. 2019;21 7:1611-20. doi:10.1038/s41436-018-0380-2.
23. Gilman P, Janzou S, Guittet D, Freeman J, DiOrio N, Blair N, et al. *PySAM (Python Wrapper for System Advisor Model" SAM")*. 2019. National Renewable Energy Lab.(NREL), Golden, CO (United States).
24. Peiffer DA, Le JM, Steemers FJ, Chang W, Jenniges T, Garcia F, et al. High-resolution genomic profiling of chromosomal aberrations using Infinium whole-genome genotyping. Genome Res. 2006;16 9:1136-48. doi:10.1101/gr.5402306.
25. Loh PR, Genovese G, Handsaker RE, Finucane HK, Reshef YA, Palamara PF, et al. Insights into clonal haematopoiesis from 8,342 mosaic chromosomal alterations. Nature. 2018;559 7714:350-5. doi:10.1038/s41586-018-0321-x.
26. Boeva V, Popova T, Bleakley K, Chiche P, Cappo J, Schleiermacher G, et al. Control-FREEC: a tool for assessing copy number and allelic content using next-generation sequencing data. Bioinformatics. 2012;28 3:423-5.
27. Zhu M, Need AC, Han Y, Ge D, Maia JM, Zhu Q, et al. Using ERDS to infer copy-number variants in high-coverage genomes. The American Journal of Human Genetics. 2012;91 3:408-21.
28. Auton A, Brooks LD, Durbin RM, Garrison EP, Kang HM, Korbel JO, et al. A global reference for human genetic variation. Nature. 2015;526 7571:68-74. doi:10.1038/nature15393.
29. OmniTier: https://www.omnitier.com/compstor-novos.
30. Gonzalez-Pena V, Natarajan S, Xia Y, Klein D, Carter R, Pang Y, et al. Accurate genomic variant detection in single cells with primary template-directed amplification. Proceedings of the National Academy of Sciences. 2021;118 24.
31. Hunter JD. Matplotlib: A 2D graphics environment. Computing in science & engineering. 2007;9 3:90-5.
32. Suvakov M, Panda A, Diesh C, Holmes I and Abyzov A. Supporting data for "CNVpytor: a tool for CNV detection and analysis from read depth and allele imbalance in whole genome sequencing" GigaScience Database. 2021; doi:http://doi.org/10.5524/100938.

Figure 1 Click here to access/download;Figure;Figure1.png

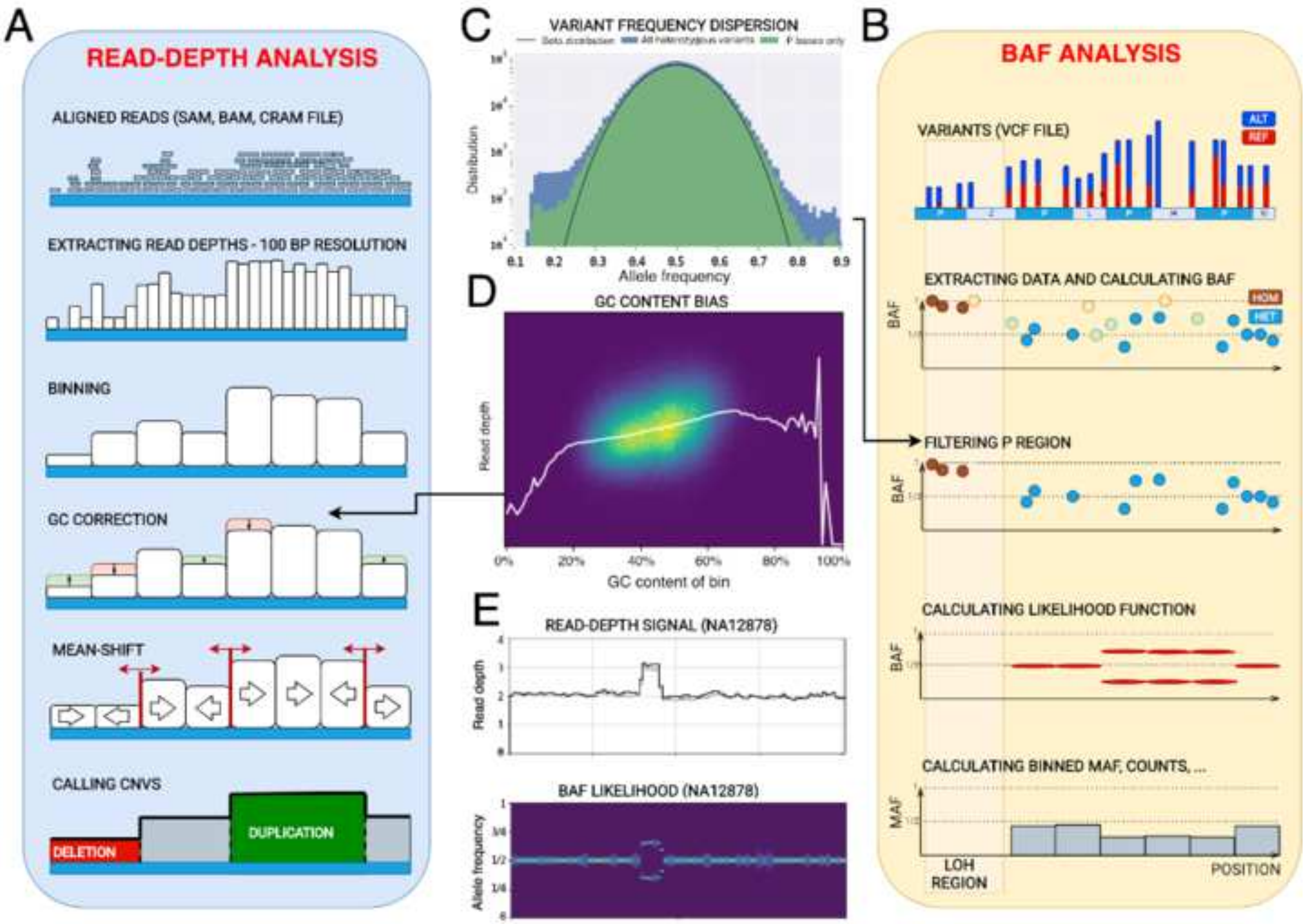

Figure 2

Click here to access/download;Figure;Figure2.png

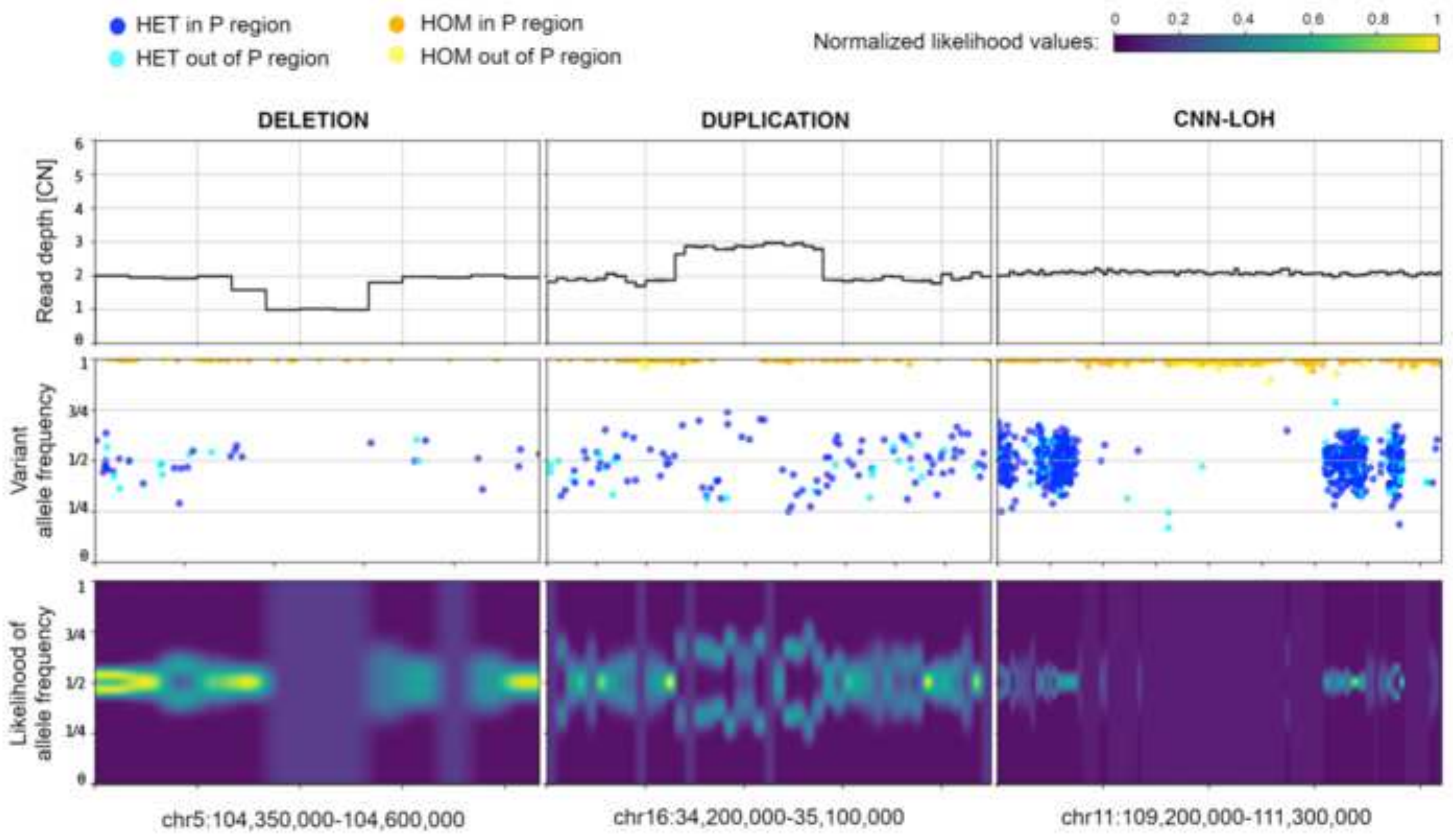

Figure 3

Click here to access/download;Figure;Figure3.png

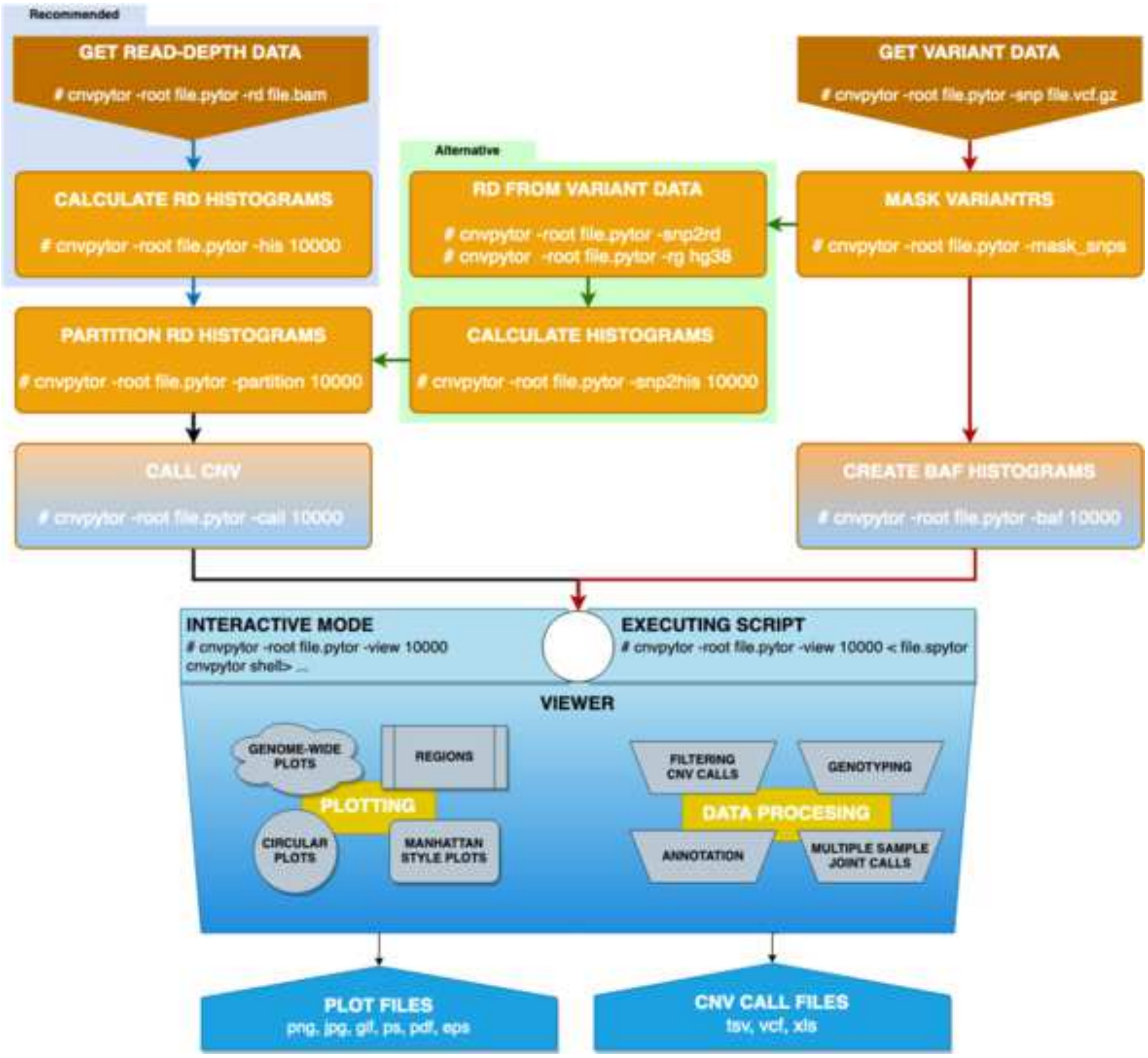

Figure 4

Click here to access/download;Figure;Figure4.png

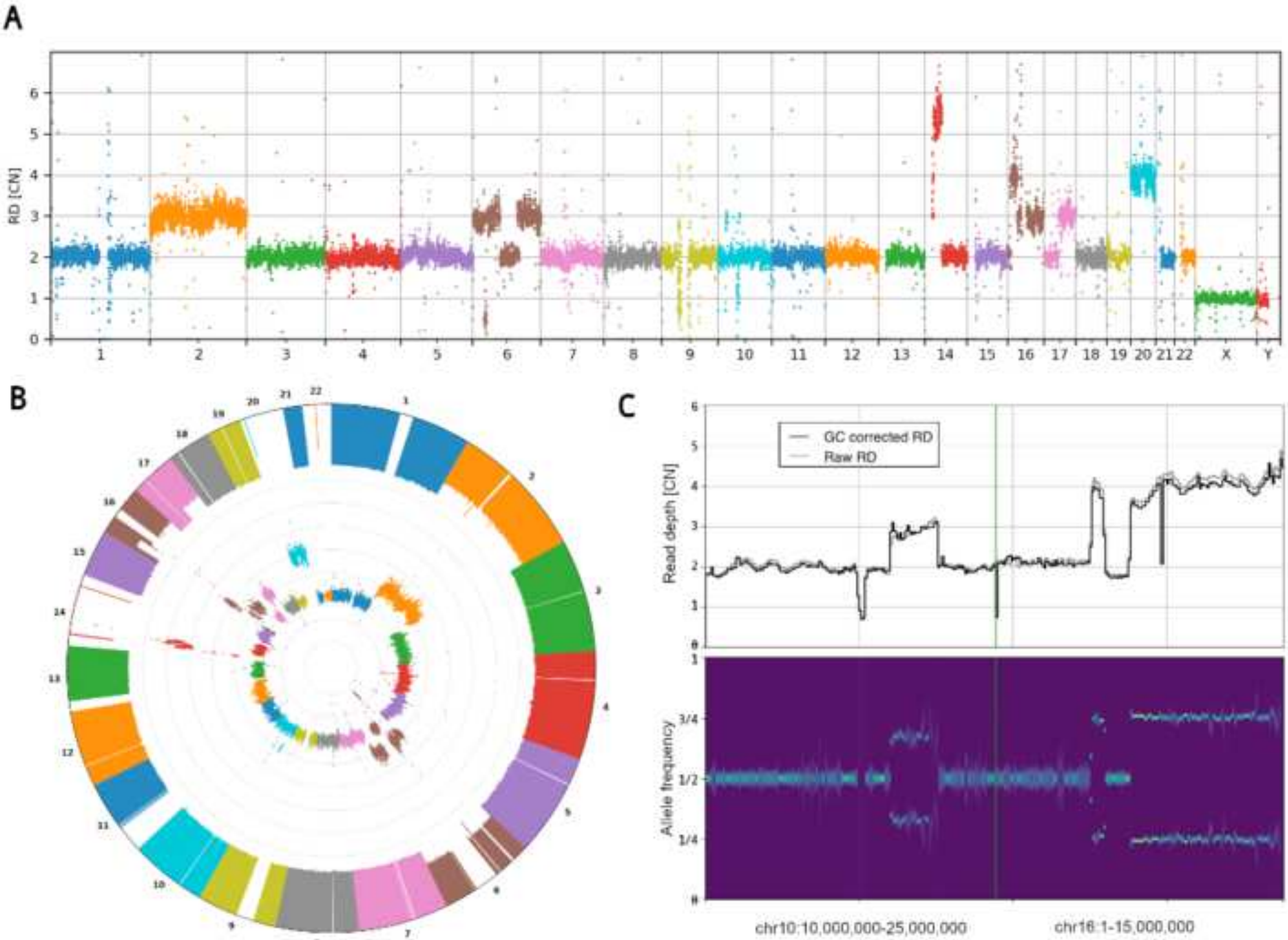

Figure S1 Supplementary material

Click here to access/download;Figure;Figure_S1_Supplemetary_material.png

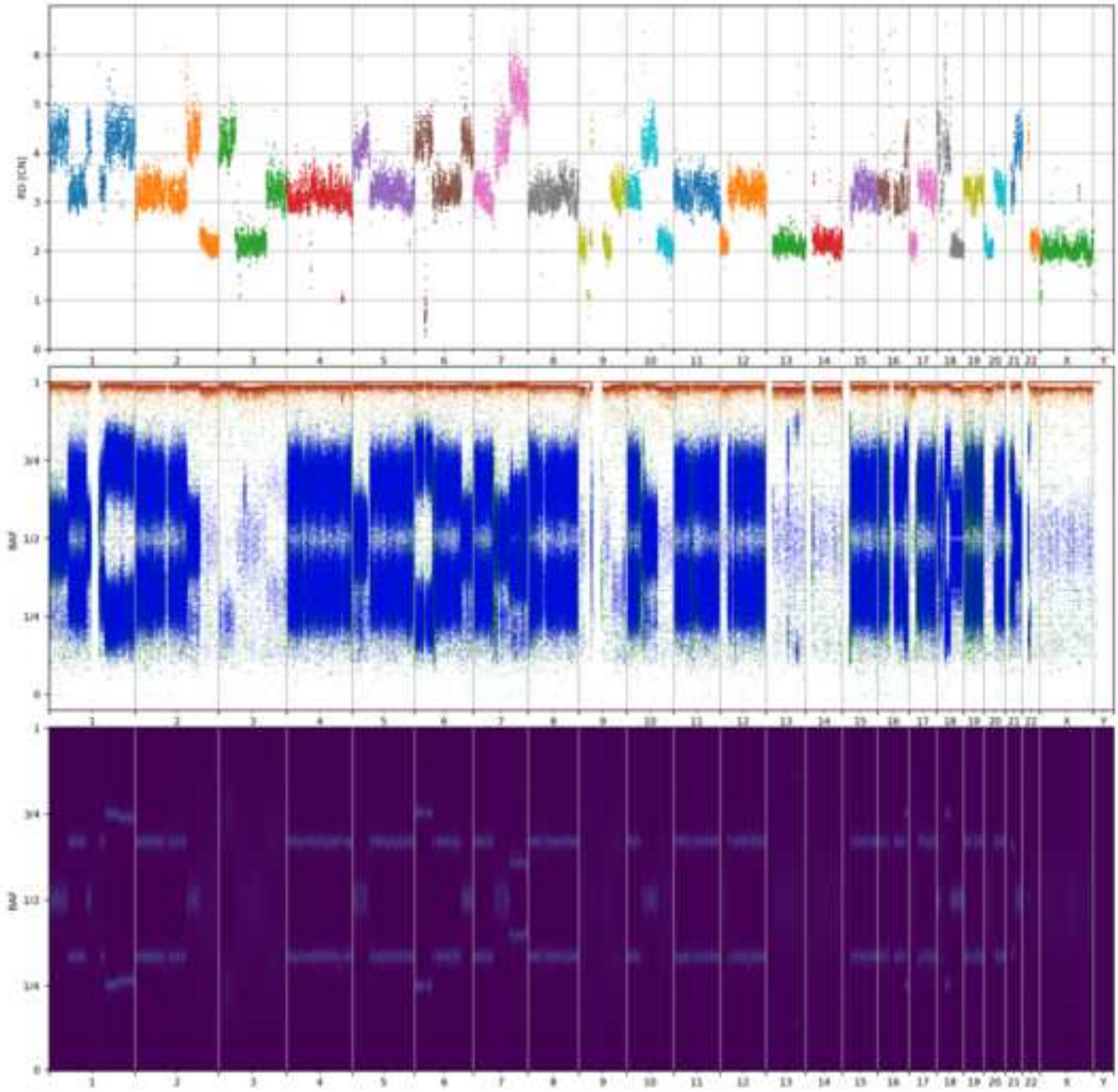

Click here to access/download;Figure;Figure_S2_Supplemetary_material.png

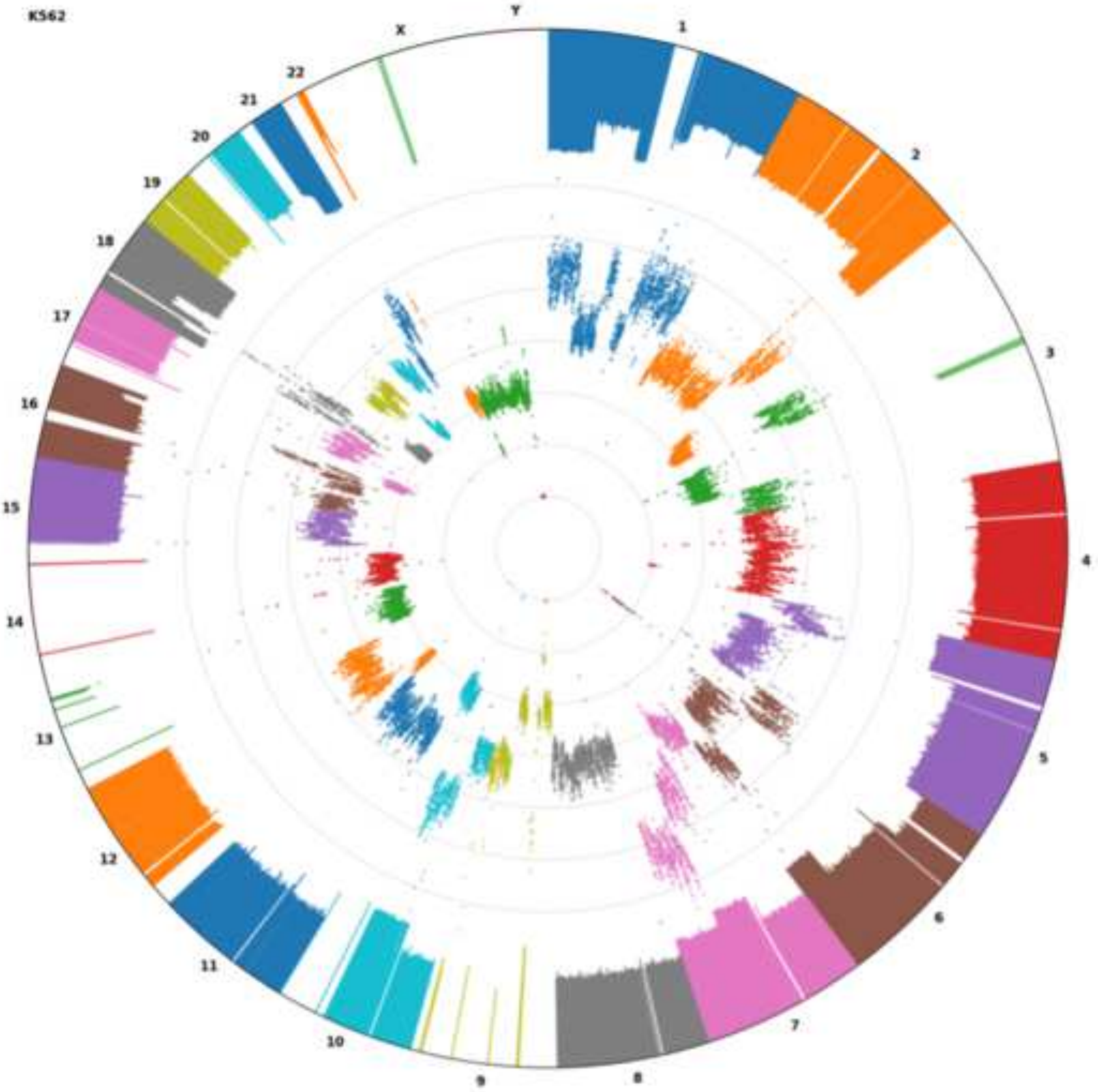

Figure S3 Supplementary material

Click here to access/download;Figure;Figure_S3_Supplemetary_material.png

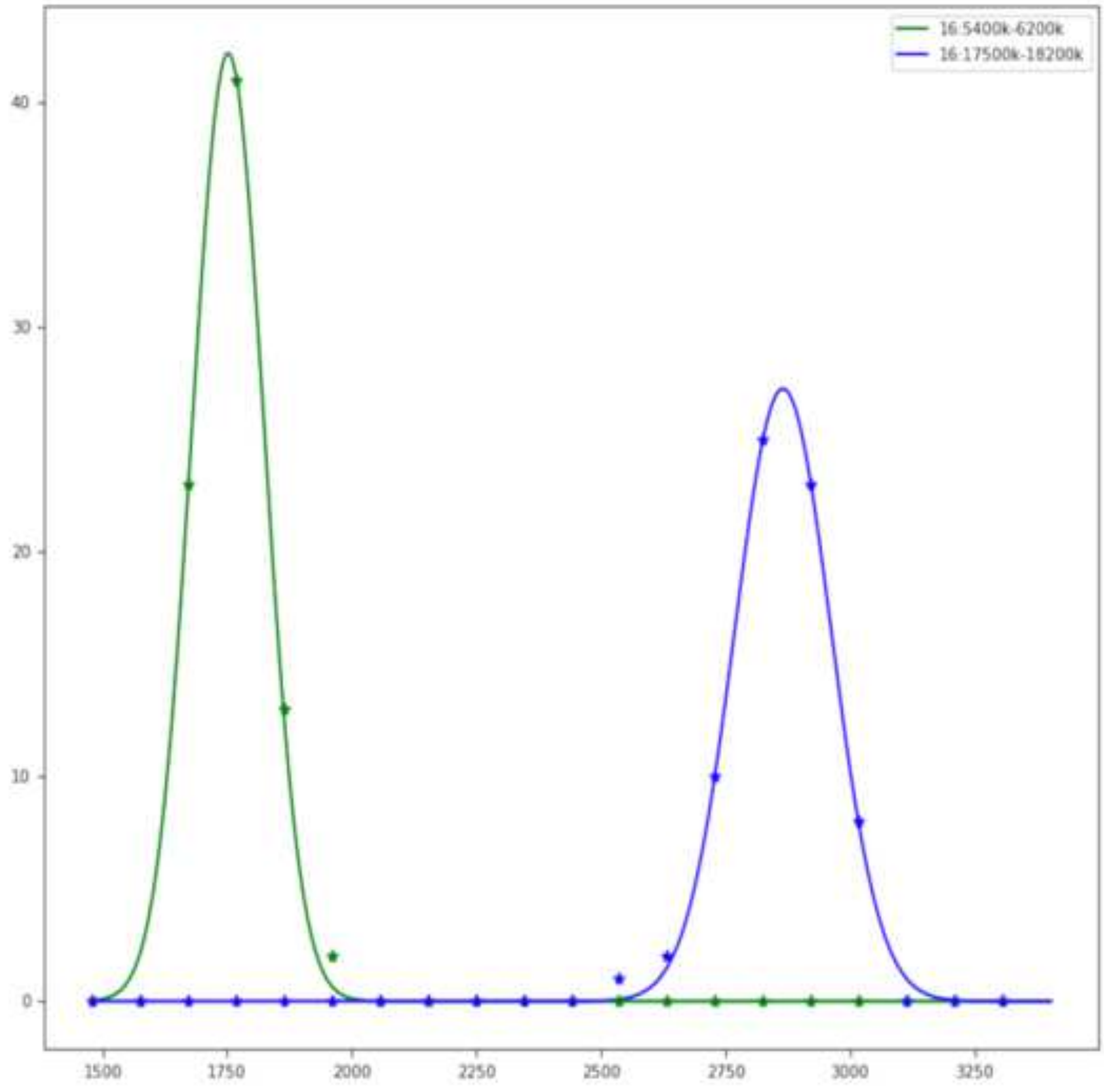

Figure S4 Supplementary material

Click here to access/download;Figure;Figure_S4_Supplemetary_material.png

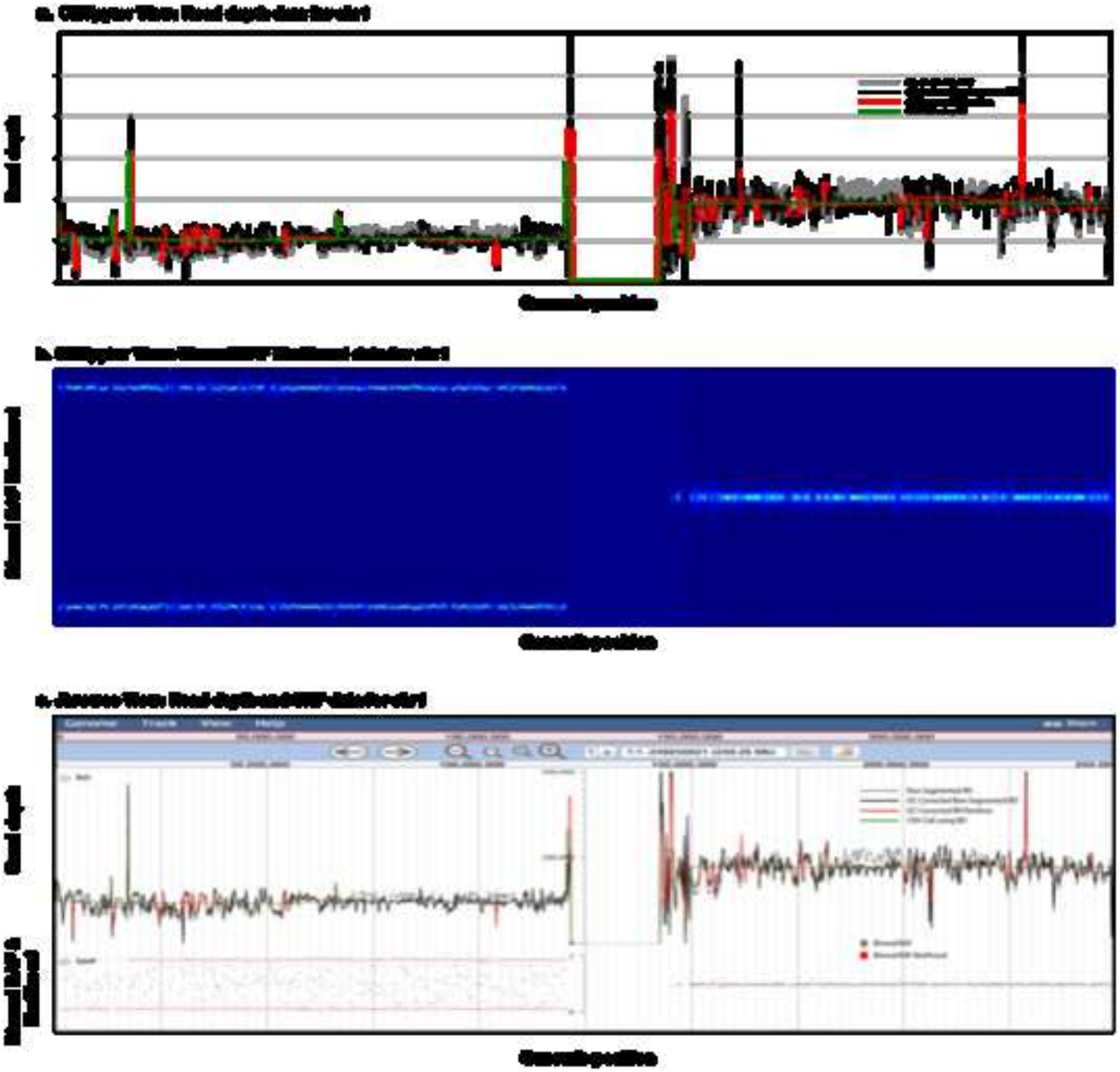

Figure S5 Supplementary material

Click here to access/download;Figure;Figure_S5_Supplemetary_material.png

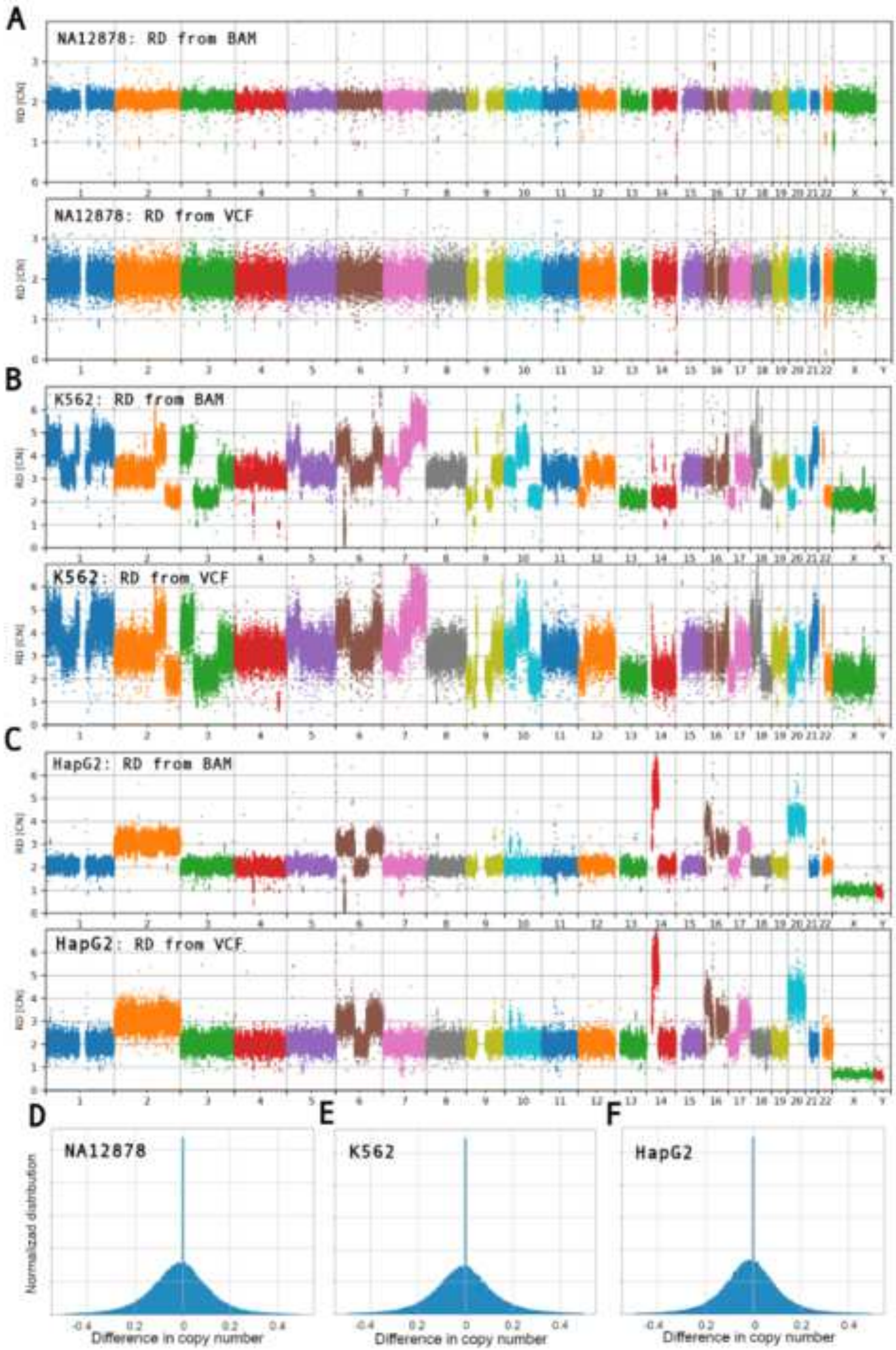

Figure S6 Supplementary material

Click here to access/download;Figure;Figure_S6_Supplemetary_material.png

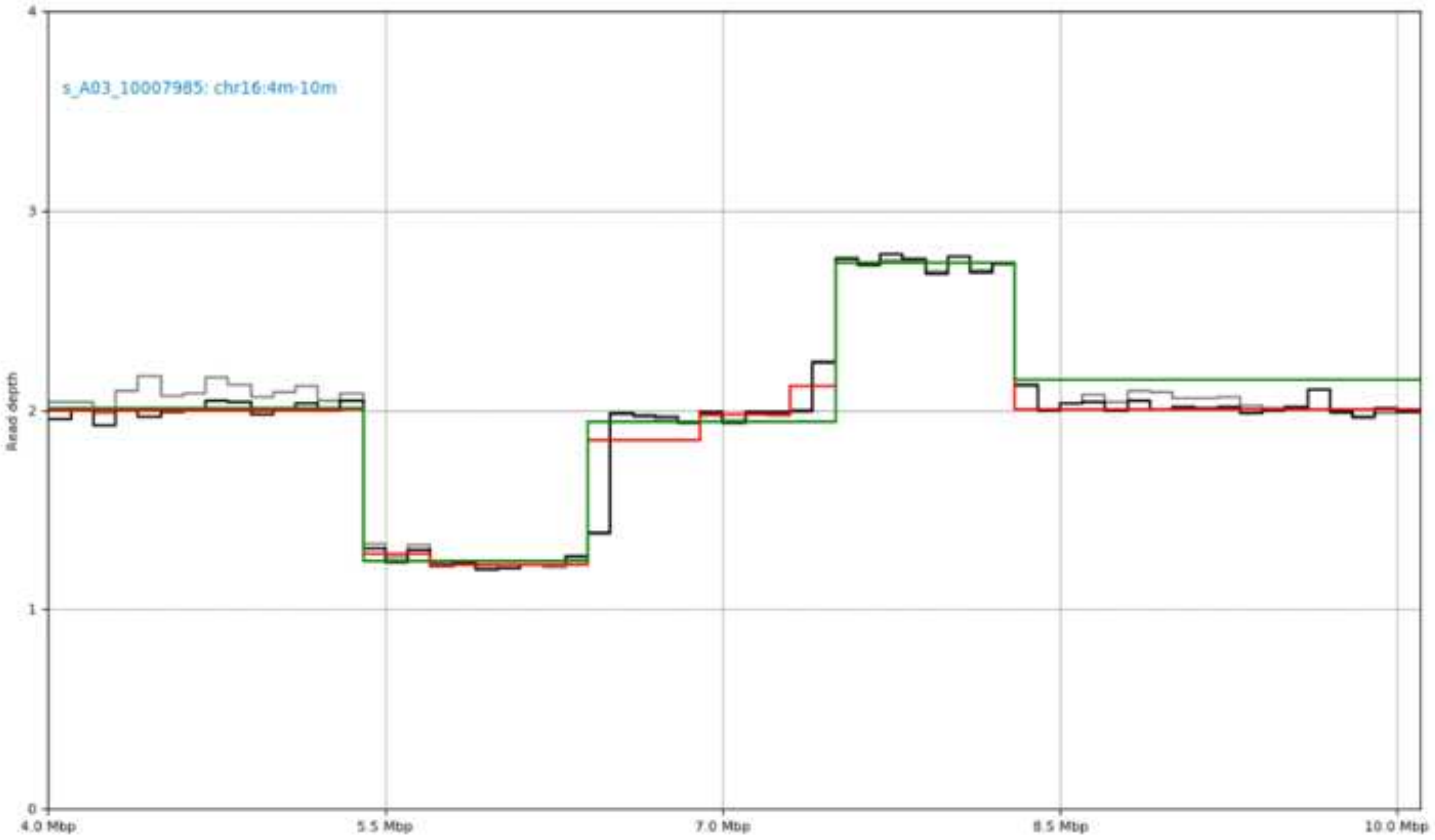

Click here to access/download
Supplementary Material
CNVpytor_Supplementary_material.docx
