# Supplementary material for: CNVpytor: a tool for copy number variation detection and analysis from read depth and allele imbalance in whole-genome sequencing
Source: Gigascience. 2021 Nov 18;10(11):giab074. doi: 10.1093/gigascience/giab074 (PMC8612020; doi:10.1093/gigascience/giab074)
Supplement: giab074_Supplemental_Files [file giab074_supplemental_files.zip › CNVpytor_Supplementary_material.docx]

**Supplemental Materials**

This PDF file includes:

- Methods – Figure preparations
- Supplemental Figure S1 - S6
- Supplemental Table

## Methods – Figure preparations

**Manuscript Figure 1**

Schematics on panels A and B are made using draw.io.

Panels C, D are plotted using matplotlib python package.

Panel E is generated using CNVpytor using procedure described in example: <https://github.com/abyzovlab/CNVpytor/blob/master/examples/region.md>

**Manuscript Figure 2 is generated using CNVpytor using procedure described in example:** <https://github.com/abyzovlab/CNVpytor/blob/master/examples/region.md>

**Manuscript Figure 3 is made using draw.io.**

**Manuscript Figure 4 is generated using CNVpytor using procedures described in examples:**A <https://github.com/abyzovlab/CNVpytor/blob/master/examples/manhattan.md>

B <https://github.com/abyzovlab/CNVpytor/blob/master/examples/circular.md>

C <https://github.com/abyzovlab/CNVpytor/blob/master/examples/region.md>

**Supplemental Figure 1 is generated using CNVpytor using procedure described in example:**

<https://github.com/abyzovlab/CNVpytor/blob/master/examples/region.md>

**Supplemental Figure 2 is generated using CNVpytor using procedure described in example:**

<https://github.com/abyzovlab/CNVpytor/blob/master/examples/circular.md>

**Supplemental Figure 3 is generated using CNVpytor using procedure described in example:**

<https://github.com/abyzovlab/CNVpytor/blob/master/examples/compare.md>

**Supplemental Figure 4 is snapshot of the browser using JBrowse with CNVpytor exported data.**

**Supplemental Figure 5 is generated using CNVpytor using procedure described in example:**

<https://github.com/abyzovlab/CNVpytor/blob/master/examples/manhattan.md>

Panels D, E, F are plotted using matplotlib python package.

**Supplemental Figure 6 is generated using CNVpytor using procedure described in example:**

<https://github.com/abyzovlab/CNVpytor/blob/master/examples/region.md>

## Supplemental Figures


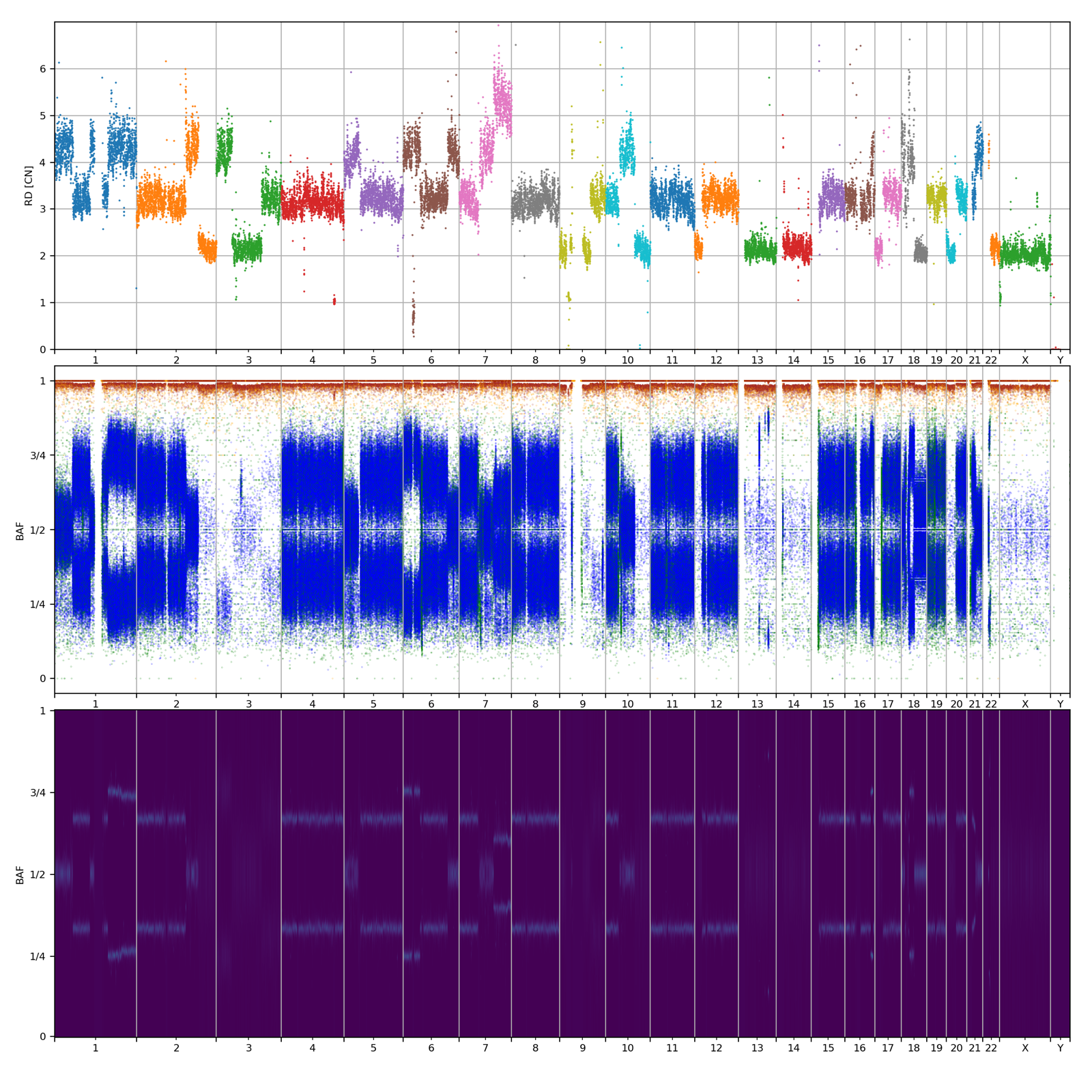


Supplemental Figure 1. Genome wide plot for K562 cell line: normalized read depth (top), B-allele frequency of individual SNPs (middle) and BAF likelihood function (bottom). Bin size is 100 kbp.


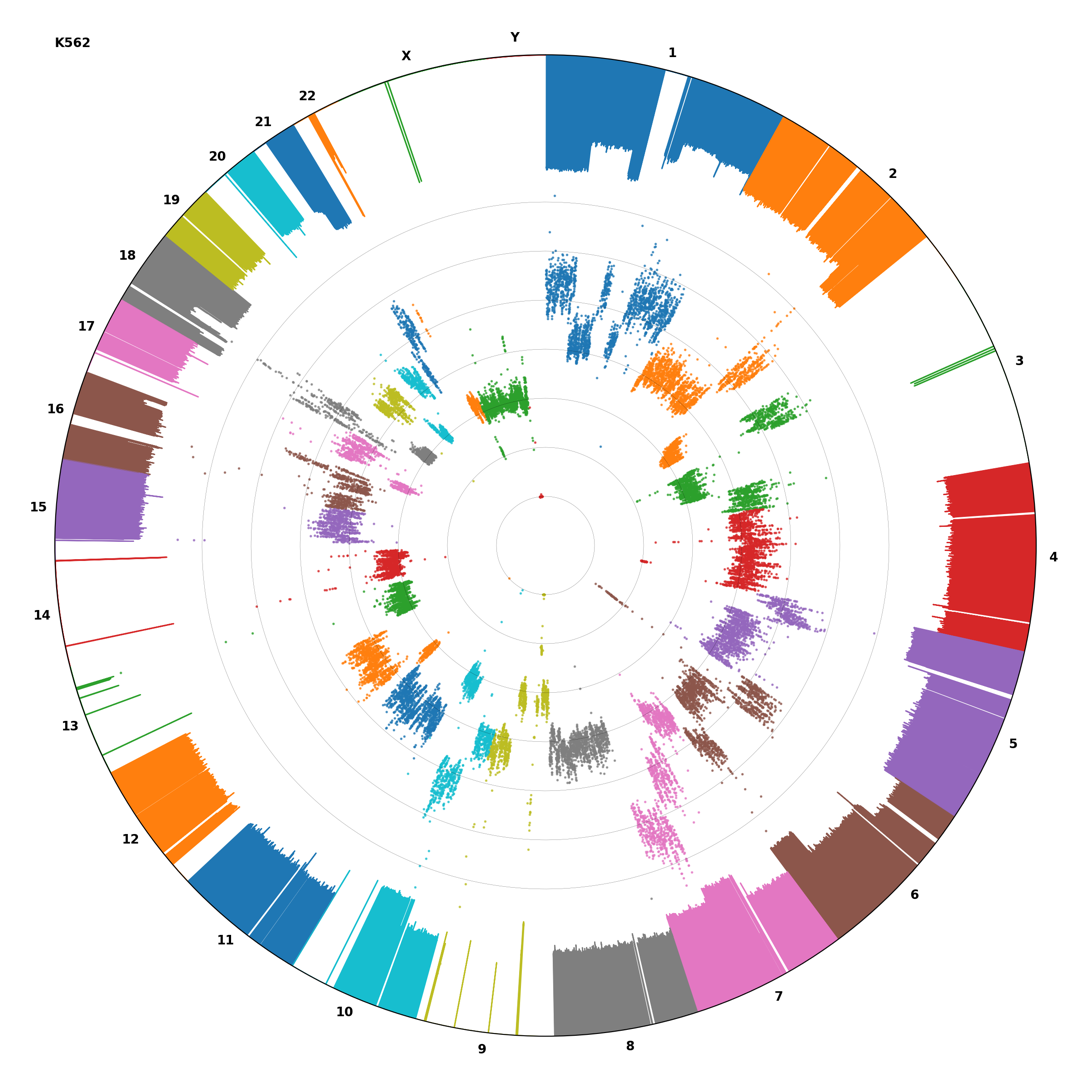
 Supplemental Figure 2. Circular plot for K562 cell line. Inner circle corresponds to read depth, outer – to binned MAF. Bin size is 100 kbp.


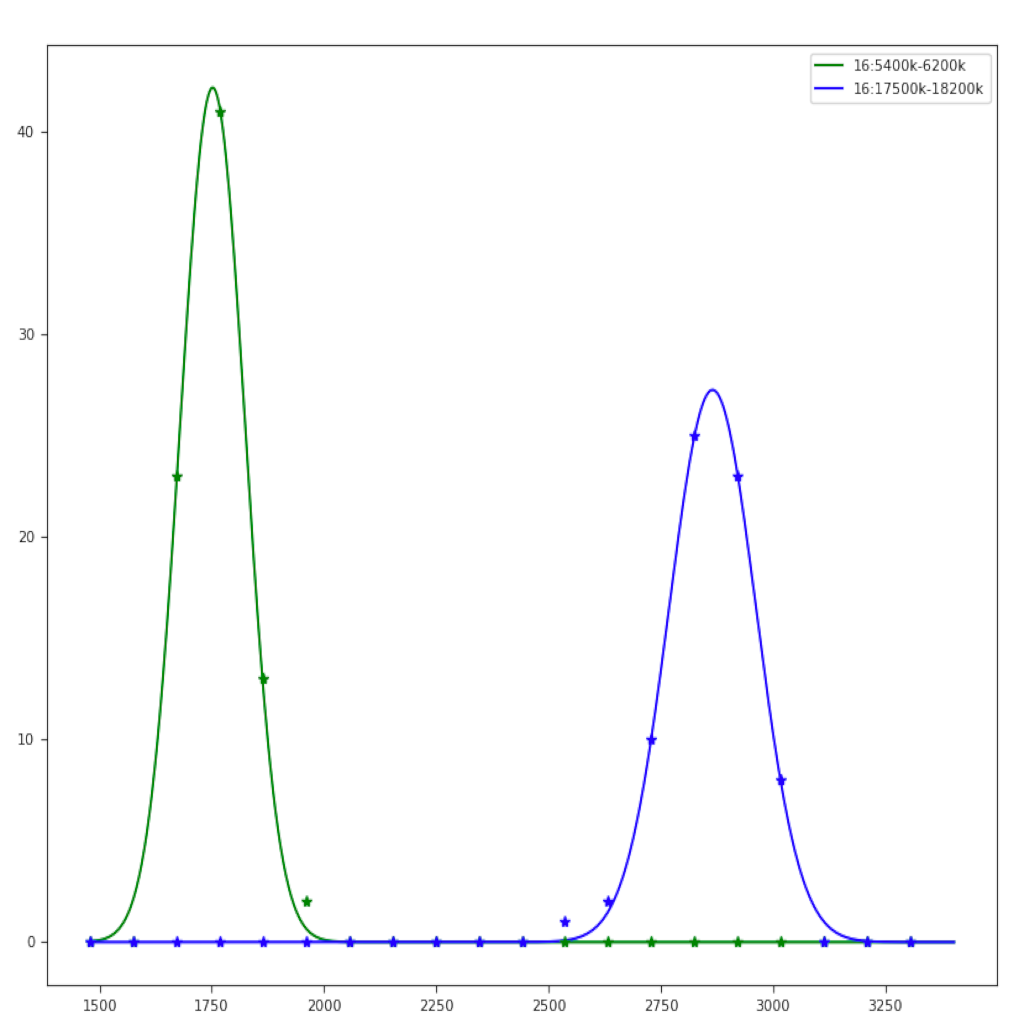


Supplemental Figure 3. Comparison of read depth statistics between two regions.


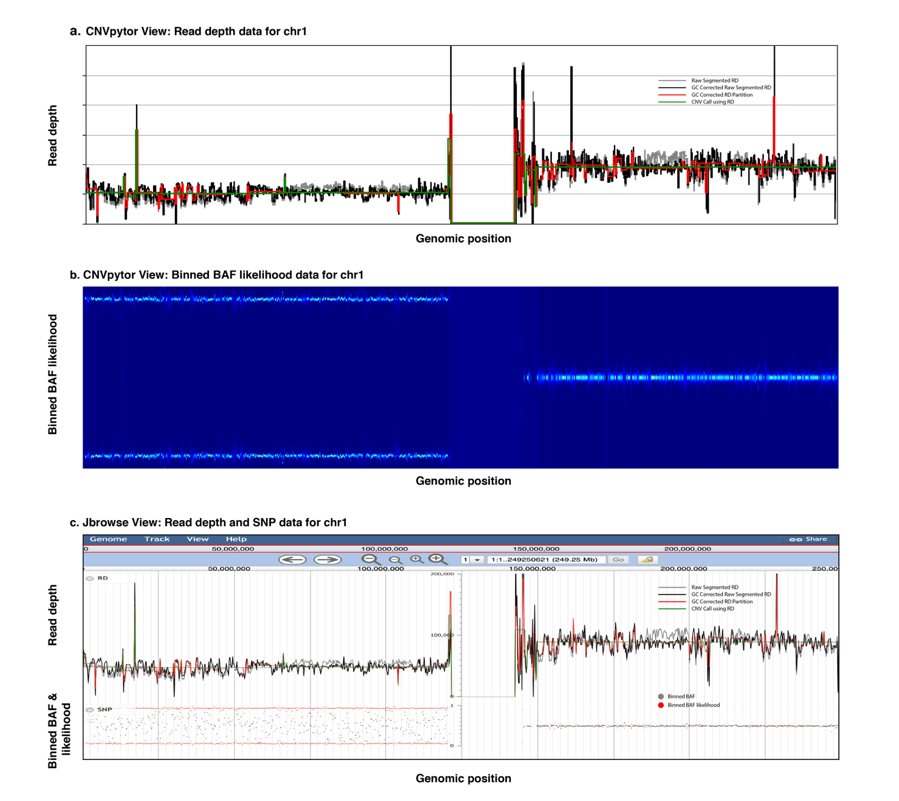


Supplemental Figure 4. JBrowse export example. (a, b) CNVpytor produced read depth and binned BAF data for a Glioblastoma cancer sample for chromosome 1 deletion is visible. (c) JBrowse view of the same data. Same color coding schema is followed here.


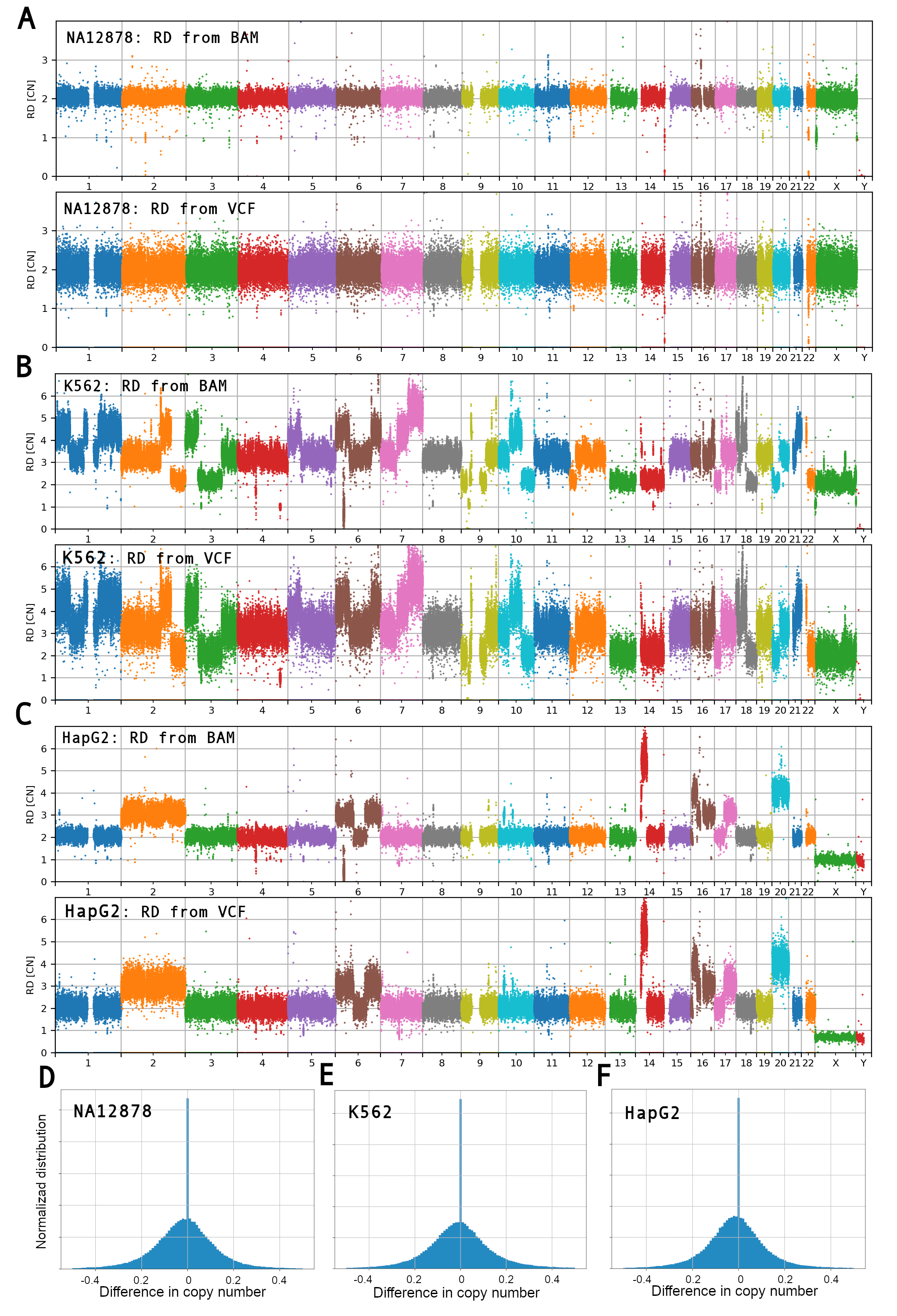


Supplemental Figure 5. Comparison between read depth signal parsed from alignment file and variant file for three samples: RD manhattan plot comparison for NA12878 sample (panel A), K562 sample (panel B) and HepG2 (panel C); distribution of differences in copy number within bins for same samples (panels D, E and F). Bin size is 10 kbp.

### School of Medicine

### School of Medicine


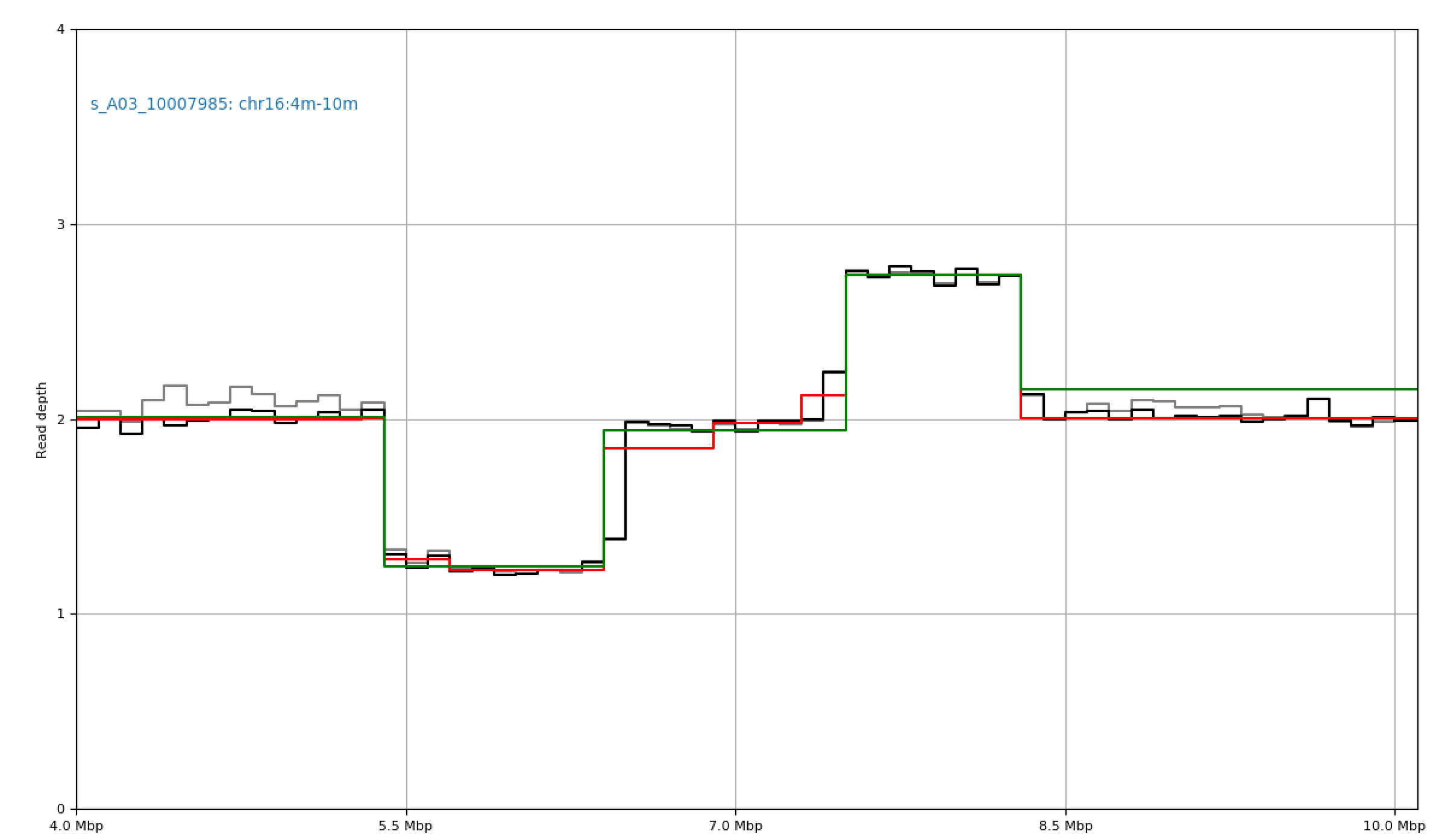


Supplemental Figure 6. CNVpytor application on polyp subclonal CNA (Kim et al., Oncotarget, 2017, PMID: 29467928). Raw RD signal is in grey, GC corrected RD signal is in black, segmentation is in red and CNA calls in green.

### School of Medicine

## Supplemental Tables

*Supplemental Table 1. Recommended minimal bin size for given coverage to ensure relative deviation od RD signal smaller then 10% for 150 base pair reads. For 100 base pair reads one can use 33% smaller bin size.*

| **Coverage** | **Minimum bin size** |
| --- | --- |
| 0.1X | 150000 |
| 0.5X | 30000 |
| 5X | 3000 |
| 30X | 500 |
| 100X | 200 |

|  | | **Control-FREEC** | **ERDS** | **Sequenza** | **CNVkit** | **Canvas** | **CopywriteR** | **GATK4 CNV** | **cn.MOPS** | **CNspector** | **CNVnator** | **CNVpytor** |
| --- | --- | --- | --- | --- | --- | --- | --- | --- | --- | --- | --- | --- |
| **Programing language** | | C++ | Perl/C | Python/R | Python | C# | R | Java | R | R | C++ | Python |
| **Installation** | | make | make | pip | pip | .NET | Bioconductor | jar | Bioconductor | git clone | make | pip |
| **Input data** | | bam/pileup | bam/vcf | bam/vcf | bam | bam | bam | bam | bam | txt/bed | bam/vcf | bam/vcf |
| **Visualization** | **RD** | YES | NO | YES | YES | NO | YES | NO | YES | YES | YES | YES |
|  | **BAF** | YES | NO | YES | NO | NO | NO | NO | YES | YES | YES | YES |
|  | **Multiple samples/regions** | YES | NO | NO | NO | NO | YES | NO | YES | YES | YES | YES |
| **Calling CNVs** | | YES | YES | YES | YES | YES | YES | YES | YES | NO | YES | YES |
| **GC correction** | | YES | YES | YES | YES | YES | YES | YES | YES | N/A | YES | YES |
| **Copy number value** | | float | float | integer | float | float | float | float | integer | float | float | float |
| **Genotyping** | | YES | NO | YES | YES | NO | NO | NO | NO | N/A | YES | YES |
| **Merging calls over multiple samples** | | NO | NO | NO | NO | YES | YES | YES | YES | N/A | NO | YES |
| **Filtering calls** | | NO | NO | NO | NO | NO | NO | NO | NO | N/A | NO | YES |
| **API / library** | | NO | NO | YES | NO | NO | YES | NO | YES | YES | NO | YES |
| **Web plugin** | | NO | NO | ­­­­­NO | NO | NO | NO | NO | NO | YES | NO | YES |

Supplemental Table 2. Comparison between CNVpytor features with other similar tools

### School of Medicine
