# Supplementary figures and images for: CNVpytor: a tool for copy number variation detection and analysis from read depth and allele imbalance in whole-genome sequencing
Source: Gigascience. 2021 Nov 18;10(11):giab074. doi: 10.1093/gigascience/giab074 (PMC8612020; doi:10.1093/gigascience/giab074)

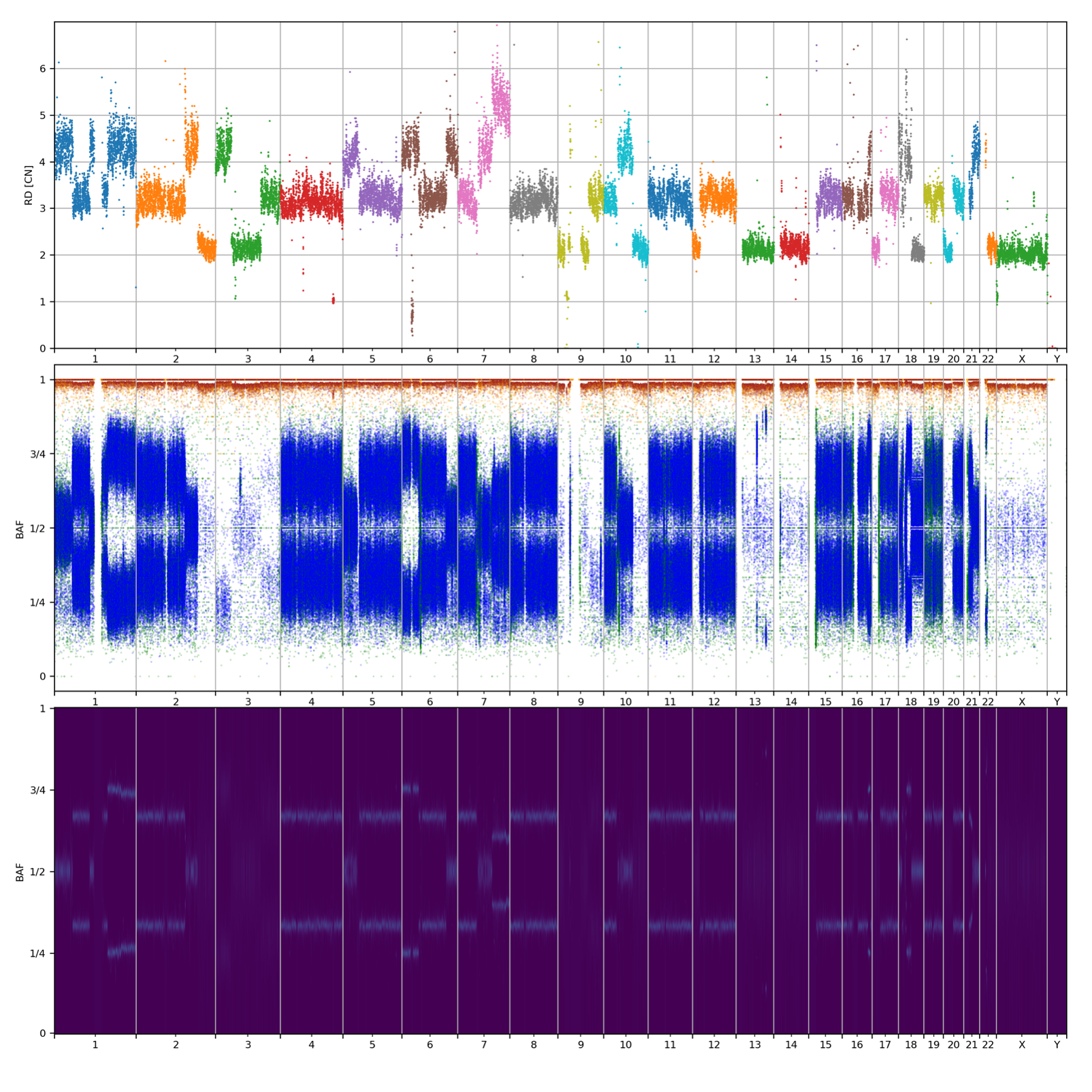

Supplement: giab074_Supplemental_Files [file giab074_supplemental_files.zip › Figure_S1_Supplemetary_material.png]

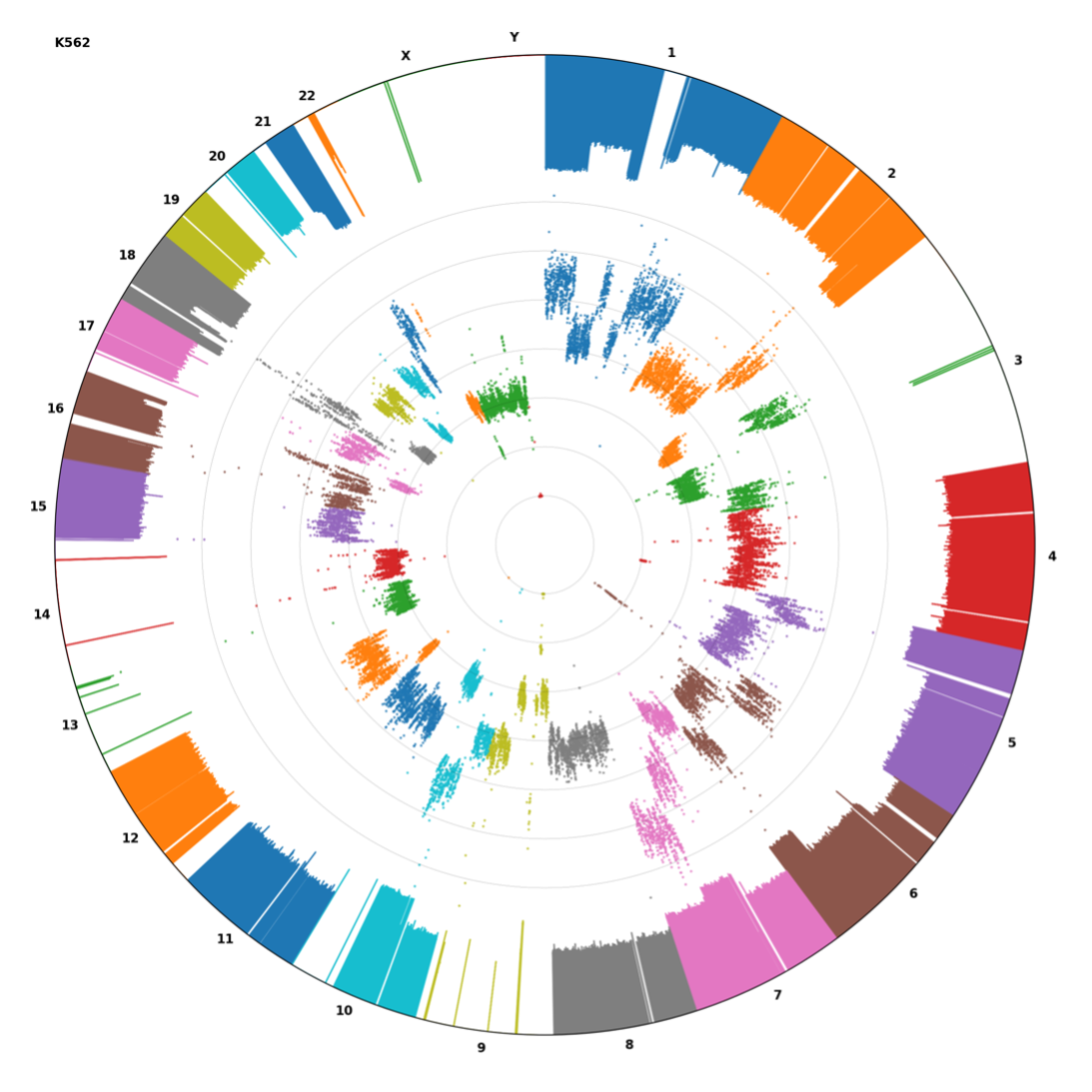

Supplement: giab074_Supplemental_Files [file giab074_supplemental_files.zip › Figure_S2_Supplemetary_material.png]

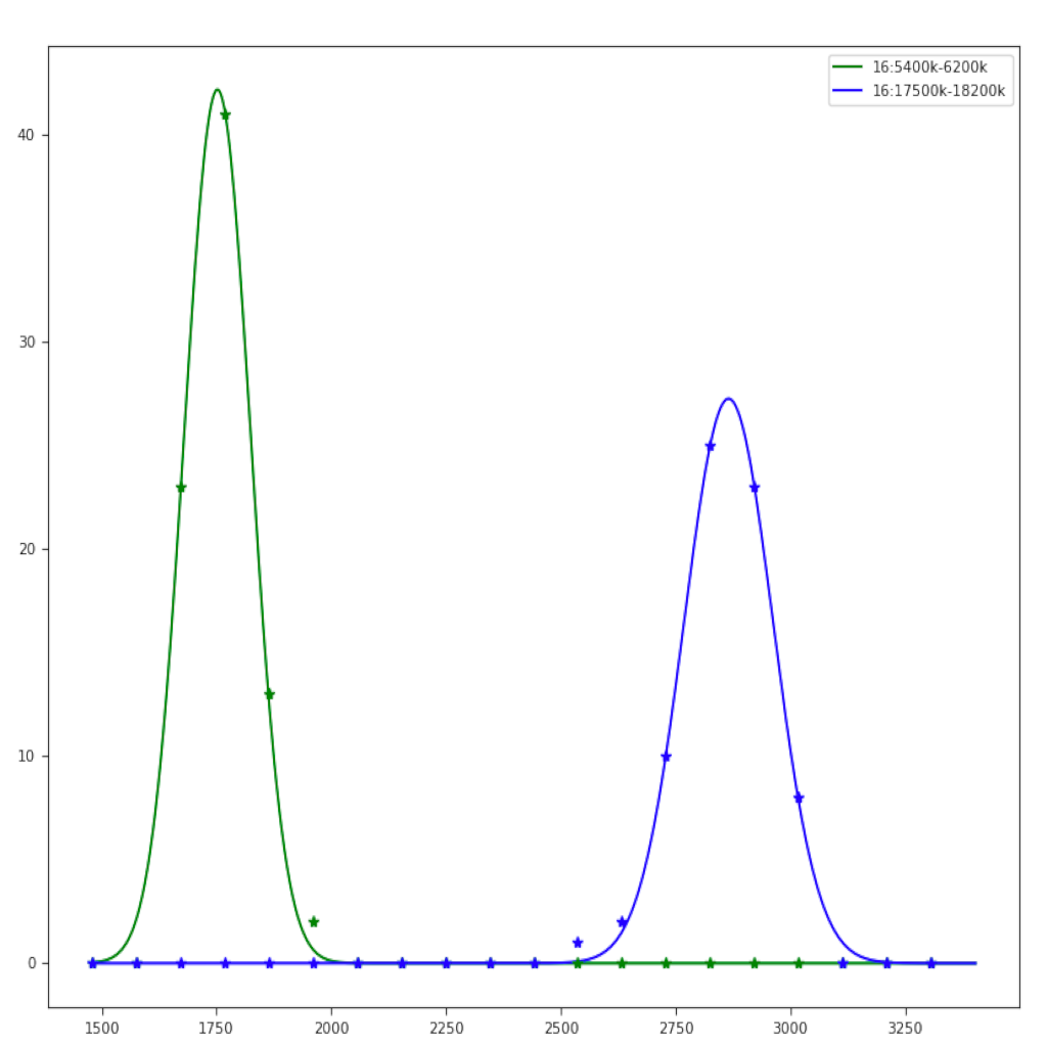

Supplement: giab074_Supplemental_Files [file giab074_supplemental_files.zip › Figure_S3_Supplemetary_material.png]

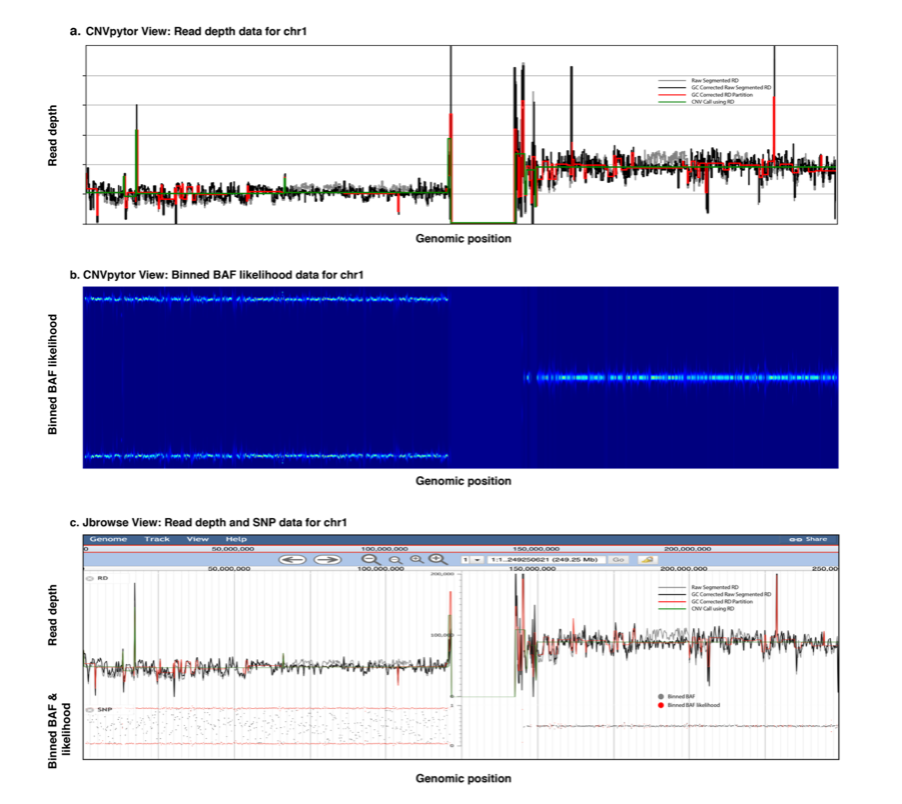

Supplement: giab074_Supplemental_Files [file giab074_supplemental_files.zip › Figure_S4_Supplemetary_material.png]

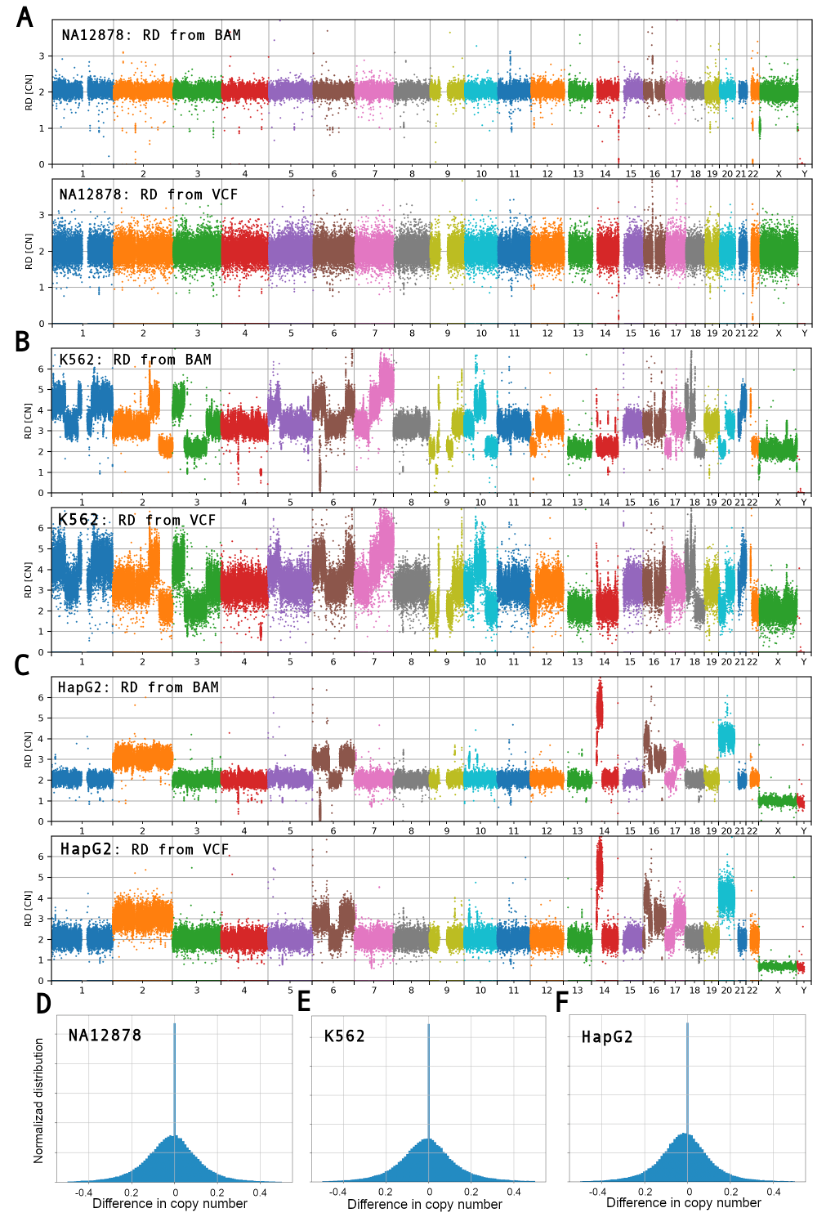

Supplement: giab074_Supplemental_Files [file giab074_supplemental_files.zip › Figure_S5_Supplemetary_material.png]

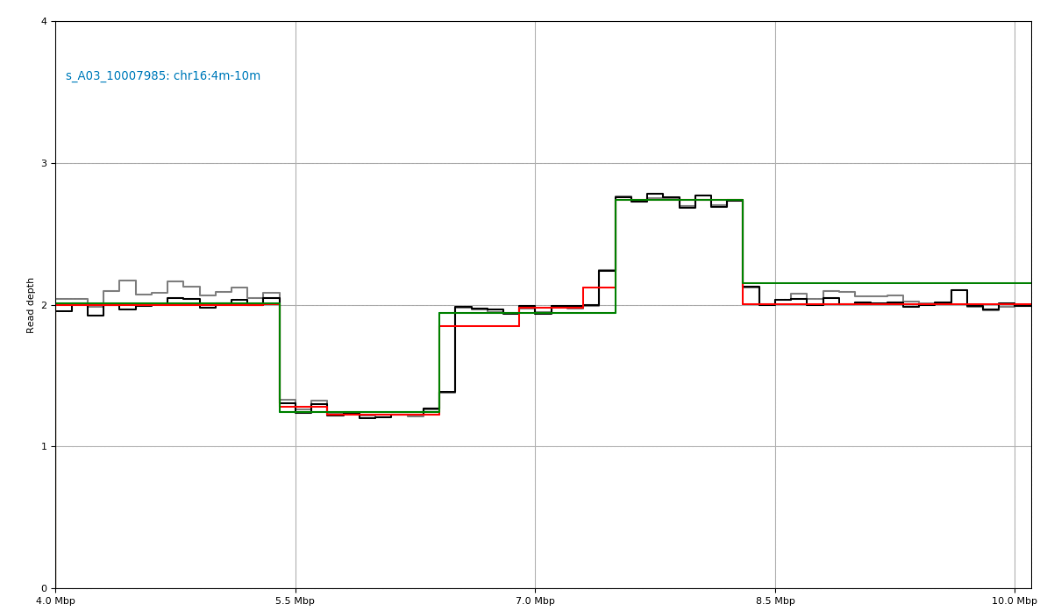

Supplement: giab074_Supplemental_Files [file giab074_supplemental_files.zip › Figure_S6_Supplemetary_material.png]
