# Supplementary material for: CNVpytor: a tool for copy number variation detection and analysis from read depth and allele imbalance in whole-genome sequencing
Source: Gigascience. 2021 Nov 18;10(11):giab074. doi: 10.1093/gigascience/giab074 (PMC8612020; doi:10.1093/gigascience/giab074)
Supplement: giab074_Response_to_Reviewer_Comments_Original_Submission [file giab074_response_to_reviewer_comments_original_submission.pdf]

Author's Response To Reviewer Comments

Close

Please fine point by point response in one of the supplementary files.

Close
