# Supplementary material for: CNVpytor: a tool for copy number variation detection and analysis from read depth and allele imbalance in whole-genome sequencing
Source: Gigascience. 2021 Nov 18;10(11):giab074. doi: 10.1093/gigascience/giab074 (PMC8612020; doi:10.1093/gigascience/giab074)
Supplement: giab074_Response_to_Reviewer_Comments_Revision_1 [file giab074_response_to_reviewer_comments_revision_1.pdf]

Author's Response To Reviewer Comments

Close

Dear Dr. Hans Zauner,

Please find resubmitted manuscript titled “CNVpytor: a tool for CNV detection and analysis from read depth and allele imbalance in whole genome sequencing” along with point-by-point response letter, attached as separate file "Response Letter.docx".

We addressed all referee concerns and critiques, particularly, as you suggested, we mentioned new functionalities and availability of prototype caller. We changed the manuscript title as the second referee suggested.

We thank you for your consideration and look forward to hearing from you.

Sincerely,

Alexej Abyzov, Ph.D.
Senior Associate Consultant
Associate Professor of Biomedical Informatics

Close
