# Supplementary material for: CNVpytor: a tool for copy number variation detection and analysis from read depth and allele imbalance in whole-genome sequencing
Source: Gigascience. 2021 Nov 18;10(11):giab074. doi: 10.1093/gigascience/giab074 (PMC8612020; doi:10.1093/gigascience/giab074)
Supplement: giab074_Response_to_Reviewer_Comments_Revision_2 [file giab074_response_to_reviewer_comments_revision_2.pdf]

Author's Response To Reviewer Comments

Close

We have addressed suggested editorial points:
- Supplemental material is updated.
- biotools ID is updated.
- GigaDB dataset is added in references and cited from "data availability" paragraph.

Close
