# Supplementary material for: CNVpytor: a tool for copy number variation detection and analysis from read depth and allele imbalance in whole-genome sequencing
Source: Gigascience. 2021 Nov 18;10(11):giab074. doi: 10.1093/gigascience/giab074 (PMC8612020; doi:10.1093/gigascience/giab074)
Supplement: giab074_Reviewer_1_Report_Original_Submission — Whitney Whitford, PhD -- 6/13/2021 Reviewed [file giab074_reviewer_1_report_original_submission.pdf]

Reviewer Report

Title: CNVpytor: a tool for CNV detection and analysis from read depth and allele imbalance in whole genome sequencing

Version: Original Submission **Date:** 6/13/2021

Reviewer name: Whitney Whitford, PhD

Reviewer Comments to Author:

This Technical Note presents an updated version of CNVnator implemented in Python called CNVpytor. CNVnator is one of the most used pieces of copy number variant (CNV) detection software, having been used by The Genome Aggregation Database (gnomAD), among others. The authors reported increased functionality of CNVpytor over CNVnator with easy to use visualisation options and more rapid analysis. The additional functionality and thorough installation and user guides available via the linked github repository and videos found online make this an extremely accessible piece of software, such that I believe it will help to further the use of next generation sequencing and CNV detection in genetic research. The authors also included an additional quantitative measure of the probability of the presence of a CNV at the called coordinates by levering the B allele frequencies (BAF) across the CNV. This is calculated using symmetrized beta function for likelihood, calculated for each bin. I agree that BAF is a crucial piece of information that has been currently underused in CNV detection and use it in my own work. The manuscript overall is a well written presentation of research which I believe will be well-utilised. I only have two pieces of minor feedback:1. The authors state that CNVpytor inherits the core engine of CNVnator. I assume that this means that CNVpytor performs identically in identifying CNVs. If this is so, I would like to see this explicitly stated.2. The inclusion of BAF is a significant improvement over CNVnator. An explanation of BAF and why levering this additional piece of data in CNV detection in the introduction could be of use for the reader.I recommend this Technical Note for publication in GigaScience.

Level of Interest

Please indicate how interesting you found the manuscript: Choose an item.

Quality of Written English

Please indicate the quality of language in the manuscript: Choose an item.

Declaration of Competing Interests

Please complete a declaration of competing interests, considering the following questions:

- Have you in the past five years received reimbursements, fees, funding, or salary from an organisation that may in any way gain or lose financially from the publication of this manuscript, either now or in the future?
- Do you hold any stocks or shares in an organisation that may in any way gain or lose financially from the publication of this manuscript, either now or in the future?
- Do you hold or are you currently applying for any patents relating to the content of the manuscript?
- Have you received reimbursements, fees, funding, or salary from an organization that holds or has applied for patents relating to the content of the manuscript?
- Do you have any other financial competing interests?
- Do you have any non-financial competing interests in relation to this paper?

If you can answer no to all of the above, write 'I declare that I have no competing interests' below. If your reply is yes to any, please give details below.

I declare that I have no competing interests

I agree to the open peer review policy of the journal. I understand that my name will be included on my report to the authors and, if the manuscript is accepted for publication, my named report including any attachments I upload will be posted on the website along with the authors' responses. I agree for my report to be made available under an Open Access Creative Commons CC-BY license (http://creativecommons.org/licenses/by/4.0/). I understand that any comments which I do not wish to be included in my named report can be included as confidential comments to the editors, which will not be published.

Choose an item.

To further support our reviewers, we have joined with Publons, where you can gain additional credit to further highlight your hard work (see: https://publons.com/journal/530/gigascience). On publication of this paper, your review will be automatically added to Publons, you can then choose whether or not to claim your Publons credit. I understand this statement.

Yes Choose an item.
