# Supplementary material for: CNVpytor: a tool for copy number variation detection and analysis from read depth and allele imbalance in whole-genome sequencing
Source: Gigascience. 2021 Nov 18;10(11):giab074. doi: 10.1093/gigascience/giab074 (PMC8612020; doi:10.1093/gigascience/giab074)
Supplement: giab074_Reviewer_1_Report_Revision_1 — Whitney Whitford, PhD -- 8/29/2021 Reviewed [file giab074_reviewer_1_report_revision_1.pdf]

Reviewer Report

Title: CNVpytor: a tool for CNV detection and analysis from read depth and allele imbalance in whole genome sequencing

Version: Revision 1 **Date:** 8/29/2021

Reviewer name: Whitney Whitford, PhD

Reviewer Comments to Author:

The reviewed manuscript from Suvakov et al., and associated response letters addresses the concerns of the reviewers from the first round of submission. The major concern raised by reviewer 2 regarding comparing the performance and computational usage of CNVpytor to previously available tools, I feel was adequately addressed by the authors. Another major concern raised by reviewer 2 was the claim that CNVpytor is appropriate for use with single cell sequencing data and cancer data. The authors demonstrated that some single cell sequencing techniques generate sufficient depth for CNVpytor to accurately call CNVs. Regarding CNV calling from cancer sequence data, the authors added the following to the manuscript: "Method is suitable for segmentation of RD data in the case of mosaicism or cancer samples and such CNAs will be called if cell frequency is high enough (Fig. S6)". Can they at least hypothesise as to what cell frequency may be high enough as this is not interpretable from Fig. S6?My main piece of feedback responds to a part of the review that Suvakov et al. did not address in their response. That is again from reviewer 2 who started their major comments with: "My main concern about this manuscript is that it does not have any scientific merit. It does not introduce any novel methodology in CNV calling or technical implementation. It introduces a new implementation based on already developed technologies and methodologies. To me it is more an extended version of CNVnator rather than a novel tool." I do not completely agree with this as the reduced storage usage and decreased runtime will pose of great utility for the bioinformatics community. However, the authors also refer in the manuscript and their response letter to additional functionality for cancer and mosaic cases, and incorporating B allele frequency in calling which are currently in the development process. The manuscript would be strengthened by CNVpytor with these functionalities being included as a single publication.

Level of Interest

Please indicate how interesting you found the manuscript: Choose an item.

Quality of Written English

Please indicate the quality of language in the manuscript: Choose an item.

Declaration of Competing Interests

Please complete a declaration of competing interests, considering the following questions:

- Have you in the past five years received reimbursements, fees, funding, or salary from an organisation that may in any way gain or lose financially from the publication of this manuscript, either now or in the future?
- Do you hold any stocks or shares in an organisation that may in any way gain or lose financially from the publication of this manuscript, either now or in the future?
- Do you hold or are you currently applying for any patents relating to the content of the manuscript?
- Have you received reimbursements, fees, funding, or salary from an organization that holds or has applied for patents relating to the content of the manuscript?
- Do you have any other financial competing interests?
- Do you have any non-financial competing interests in relation to this paper?

If you can answer no to all of the above, write 'I declare that I have no competing interests' below. If your reply is yes to any, please give details below.

I declare that I have no competing interests

I agree to the open peer review policy of the journal. I understand that my name will be included on my report to the authors and, if the manuscript is accepted for publication, my named report including any attachments I upload will be posted on the website along with the authors' responses. I agree for my report to be made available under an Open Access Creative Commons CC-BY license (http://creativecommons.org/licenses/by/4.0/). I understand that any comments which I do not wish to be included in my named report can be included as confidential comments to the editors, which will not be published.

Choose an item.

To further support our reviewers, we have joined with Publons, where you can gain additional credit to further highlight your hard work (see: https://publons.com/journal/530/gigascience). On publication of this paper, your review will be automatically added to Publons, you can then choose whether or not to claim your Publons credit. I understand this statement.

Yes Choose an item.
