# Supplementary material for: CNVpytor: a tool for copy number variation detection and analysis from read depth and allele imbalance in whole-genome sequencing
Source: Gigascience. 2021 Nov 18;10(11):giab074. doi: 10.1093/gigascience/giab074 (PMC8612020; doi:10.1093/gigascience/giab074)
Supplement: giab074_Reviewer_2_Report_Revision_1 — Sheida Nabavi -- 9/11/2021 Reviewed [file giab074_reviewer_2_report_revision_1.pdf]

Reviewer Report

Title: CNVpytor: a tool for CNV detection and analysis from read depth and allele imbalance in whole genome sequencing

Version: Revision 1 **Date:** 9/11/2021

Reviewer name: Sheida Nabavi

Reviewer Comments to Author:

Most of my comments are addressed.As authors mentioned the proposed tool is an extension of their previous tool (the main algorithm for CNV detection is the same). Since the novelty and scientific merit is not significant, using a new name for the tool rather than introducing a new version of the old one is confusing. It is better authors name the tool and introduce it as a new version, specially that the old tool is one of the commonly used CNV detection tool.In the response authors stated: "However, within scope of this paper we did not use BAF for detection on CNVs, only for visualization and genotyping. We are working on extension on CNVpytor that will use BAF as an additional information during calling step", Therefore using the title as "a tool for CNV/CNA detection and analysis from read depth and allele imbalance in whole genome sequencing" is misleading.Also authors added :" However, more accurate approach to cancer and mosaic cases are part of development in progress." Considering the mentioned statement, indicating "a tool for CNV/CNA detection" is not appropriate and "CNA" needs to be deleted.I recommend authors change the title. I am not convinced by the 2.3 response. All the approaches for CNV detection for single cell sequencing data are based on the read depth analysis since the coverage is very low. And they use a very large window size (more than 200K bp). But the default of the proposed tool is a window size of 100 bp which is not appropriate for single cell sequencing data. In real practice, the coverage of single cell DNA sequencing data is <1 and the statement :"Provided good amplification and high coverage detecting CNVs in single cell," is not a practical discussion about applicability of the proposed tool to the single cell sequencing data. In addition, BAF data used for visualization and genotyping in this tool (the new addition) cannot be obtained from very low coverage (<1) sequencing data. I recommend authors provide more practical discussion about the applicability of the tool for single cell sequencing data or do not mention it.

Level of Interest

Please indicate how interesting you found the manuscript: Choose an item.

Quality of Written English

Please indicate the quality of language in the manuscript: Choose an item.

Declaration of Competing Interests

Please complete a declaration of competing interests, considering the following questions:

- Have you in the past five years received reimbursements, fees, funding, or salary from an organisation that may in any way gain or lose financially from the publication of this manuscript, either now or in the future?
- Do you hold any stocks or shares in an organisation that may in any way gain or lose financially from the publication of this manuscript, either now or in the future?
- Do you hold or are you currently applying for any patents relating to the content of the manuscript?
- Have you received reimbursements, fees, funding, or salary from an organization that holds or has applied for patents relating to the content of the manuscript?
- Do you have any other financial competing interests?
- Do you have any non-financial competing interests in relation to this paper?

If you can answer no to all of the above, write 'I declare that I have no competing interests' below. If your reply is yes to any, please give details below.

I declare that I have no competing interests.

I agree to the open peer review policy of the journal. I understand that my name will be included on my report to the authors and, if the manuscript is accepted for publication, my named report including any attachments I upload will be posted on the website along with the authors' responses. I agree for my report to be made available under an Open Access Creative Commons CC-BY license (http://creativecommons.org/licenses/by/4.0/). I understand that any comments which I do not wish to be included in my named report can be included as confidential comments to the editors, which will not be published.

Choose an item.

To further support our reviewers, we have joined with Publons, where you can gain additional credit to further highlight your hard work (see: https://publons.com/journal/530/gigascience). On publication of this paper, your review will be automatically added to Publons, you can then choose whether or not to claim your Publons credit. I understand this statement.

Yes Choose an item.
