# Supplementary material for: CNVpytor: a tool for copy number variation detection and analysis from read depth and allele imbalance in whole-genome sequencing
Source: Gigascience. 2021 Nov 18;10(11):giab074. doi: 10.1093/gigascience/giab074 (PMC8612020; doi:10.1093/gigascience/giab074)
Supplement: giab074_Reviewer_3_Report_Original_Submission — Xiao Dong -- 7/5/2021 Reviewed [file giab074_reviewer_3_report_original_submission.pdf]

Reviewer Report

Title: CNVpytor: a tool for CNV detection and analysis from read depth and allele imbalance in whole genome sequencing

Version: Original Submission **Date:** 7/5/2021

Reviewer name: Xiao Dong

Reviewer Comments to Author:

In the manuscript, entitled "CNVpytor: a tool for CNV/CNA detection and analysis from read depth and alle imbalance in whole genome sequencing", Suvakov et al., present an extension of CNVnator, a popular CNV caller they previously developed, in Python with significant calculating speed improvement and a newly implemented CNV detection algorithm based on B-allele frequency. Although the current development is based on the original CNVnator, I think the new development in CNVpytor is certainly significant and important. This is not only because of the significant performance improvement and new features in the CNVpytor as pointed out already by the authors, but also because, in my opinion, it is important to re-engineer the classical bioinformatic tools, e.g., CNVnator (developed 10 years ago), with up-to-date programming languages and trim them to work efficiently under modern computational hardware and OS environment. This will save substantial amount of time and effort for their users, and therefore, be significantly useful to the field of research.I have two minor suggestions:1. What is the resolution of CNVpytor for detecting CNAs? This information would be very useful for its users. I suggest the authors at least can provide a comment on the maximum resolution that they recommend. 2. What is the minimum sequencing depth required by CNVpytor, and can it be applied to other types of DNA sequencing data, e.g., whole-exome sequencing? Same as the above, I think these information may also be very useful.

Level of Interest

Please indicate how interesting you found the manuscript: Choose an item.

Quality of Written English

Please indicate the quality of language in the manuscript: Choose an item.

Declaration of Competing Interests

Please complete a declaration of competing interests, considering the following questions:

- Have you in the past five years received reimbursements, fees, funding, or salary from an organisation that may in any way gain or lose financially from the publication of this manuscript, either now or in the future?

- Do you hold any stocks or shares in an organisation that may in any way gain or lose financially from the publication of this manuscript, either now or in the future?
- Do you hold or are you currently applying for any patents relating to the content of the manuscript?
- Have you received reimbursements, fees, funding, or salary from an organization that holds or has applied for patents relating to the content of the manuscript?
- Do you have any other financial competing interests?
- Do you have any non-financial competing interests in relation to this paper?

If you can answer no to all of the above, write 'I declare that I have no competing interests' below. If your reply is yes to any, please give details below.

I am a co-founder of SingulOmics Corp. It is a biotech company at New York City, USA. However, I do not have any financial competing interests in relation to this specific paper.

I agree to the open peer review policy of the journal. I understand that my name will be included on my report to the authors and, if the manuscript is accepted for publication, my named report including any attachments I upload will be posted on the website along with the authors' responses. I agree for my report to be made available under an Open Access Creative Commons CC-BY license (http://creativecommons.org/licenses/by/4.0/). I understand that any comments which I do not wish to be included in my named report can be included as confidential comments to the editors, which will not be published.

Choose an item.

To further support our reviewers, we have joined with Publons, where you can gain additional credit to further highlight your hard work (see: https://publons.com/journal/530/gigascience). On publication of this paper, your review will be automatically added to Publons, you can then choose whether or not to claim your Publons credit. I understand this statement.

Yes Choose an item.
