# Supplementary material for: CNVpytor: a tool for copy number variation detection and analysis from read depth and allele imbalance in whole-genome sequencing
Source: Gigascience. 2021 Nov 18;10(11):giab074. doi: 10.1093/gigascience/giab074 (PMC8612020; doi:10.1093/gigascience/giab074)
Supplement: giab074_Reviewer_3_Report_Revision_1 — Xiao Dong -- 9/1/2021 Reviewed [file giab074_reviewer_3_report_revision_1.pdf]

Reviewer Report

Title: CNVpytor: a tool for CNV detection and analysis from read depth and allele imbalance in whole genome sequencing

Version: Revision 1 **Date:** 9/1/2021

Reviewer name: Xiao Dong

Reviewer Comments to Author:

The main point of the manuscript is about an update of an existing (but important) software. The updates include new platform (python), new interface (web plugin) and functionality (merging variants from multiple samples and filtering), which are all clearly listed in the new Supplemental Table 2 with a comparison with many other existing software tools including its original tool (CNVnator). The updates also include performance improvement, which is parallel processing. Although parallel processing cannot improve accuracy, it is a very important feature because it will save its users significant amount of time.The "in depth" comparison mentioned in the discussion between one of the reviewers and the authors is about accuracy - which I agree with the authors that it is the same as the original tool as expected. However, it is clear that the bullet point of the manuscript is about all the other aspect of updates (as described above), not its accuracy. So, in my opinion, the authors have provided a detailed comparison with existing tools in the Suppl. Table 2.So, I think the current manuscript fits exactly the criteria of the journal's Technical Note, and fully support its publication.

Level of Interest

Please indicate how interesting you found the manuscript: Choose an item.

Quality of Written English

Please indicate the quality of language in the manuscript: Choose an item.

Declaration of Competing Interests

Please complete a declaration of competing interests, considering the following questions:

- Have you in the past five years received reimbursements, fees, funding, or salary from an organisation that may in any way gain or lose financially from the publication of this manuscript, either now or in the future?
- Do you hold any stocks or shares in an organisation that may in any way gain or lose financially from the publication of this manuscript, either now or in the future?
- Do you hold or are you currently applying for any patents relating to the content of the manuscript?

- Have you received reimbursements, fees, funding, or salary from an organization that holds or has applied for patents relating to the content of the manuscript?
- Do you have any other financial competing interests?
- Do you have any non-financial competing interests in relation to this paper?

If you can answer no to all of the above, write 'I declare that I have no competing interests' below. If your reply is yes to any, please give details below.

I am a co-funder of SingulOmics Corp, a biotech company at New York City. However, the company and myself have no financial relationship with any of the authors, the institution, nor the content of the manuscript.

I agree to the open peer review policy of the journal. I understand that my name will be included on my report to the authors and, if the manuscript is accepted for publication, my named report including any attachments I upload will be posted on the website along with the authors' responses. I agree for my report to be made available under an Open Access Creative Commons CC-BY license (http://creativecommons.org/licenses/by/4.0/). I understand that any comments which I do not wish to be included in my named report can be included as confidential comments to the editors, which will not be published.

Choose an item.

To further support our reviewers, we have joined with Publons, where you can gain additional credit to further highlight your hard work (see: https://publons.com/journal/530/gigascience). On publication of this paper, your review will be automatically added to Publons, you can then choose whether or not to claim your Publons credit. I understand this statement.

Yes Choose an item.
